# The Immune Landscape of Human Primary Lung Tumors Is Th2 Skewed

**DOI:** 10.3389/fimmu.2021.764596

**Published:** 2021-11-18

**Authors:** Astri Frafjord, Linn Buer, Clara Hammarström, Henrik Aamodt, Per Reidar Woldbæk, Odd Terje Brustugun, Åslaug Helland, Inger Øynebråten, Alexandre Corthay

**Affiliations:** ^1^ Tumor Immunology Lab, Department of Pathology, Rikshospitalet, Oslo University Hospital and University of Oslo, Oslo, Norway; ^2^ Department of Pathology, Rikshospitalet, Oslo University Hospital, Oslo, Norway; ^3^ Department of Cardiothoracic Surgery, Ullevål Hospital, Oslo University Hospital, Oslo, Norway; ^4^ Section of Oncology, Drammen Hospital, Vestre Viken Hospital Trust, Drammen, Norway; ^5^ Department of Genetics, Institute for Cancer Research, The Norwegian Radium Hospital, Oslo University Hospital, Oslo, Norway; ^6^ Department of Oncology, The Norwegian Radium Hospital, Oslo University Hospital, Oslo, Norway; ^7^ Institute of Clinical Medicine, University of Oslo, Oslo, Norway; ^8^ Hybrid Technology Hub – Centre of Excellence, Institute of Basic Medical Sciences, University of Oslo, Oslo, Norway

**Keywords:** CD4 T cells, cancer immunosurveillance, class of immune response, multiplex immunohistochemistry, NSCLC, Th1, Th2, Treg

## Abstract

Tumor-specific T helper (Th) cells have a central role in the immune response against cancer. However, there exist distinct Th cell subsets with very different and antagonizing properties. Some Th subsets such as Th1 protect against cancer, while others (Th2, T regulatory/Treg) are considered detrimental or of unknown significance (T follicular helper/Tfh, Th17). The Th composition of human solid tumors remains poorly characterized. Therefore, we established a four-color multiplex chromogenic immunohistochemical assay for detection of Th1, Th2, Th17, Tfh and Treg cells in human tumor sections. The method was used to analyze resected primary lung tumors from 11 patients with non-small cell lung cancer (NSCLC). Four microanatomical regions were investigated: tumor epithelium, tumor stroma, peritumoral tertiary lymphoid structures (TLS) and non-cancerous distal lung tissue. In tumor epithelium and stroma, most CD4^+^ T cells identified had either a Th2 (GATA-3^+^CD3^+^CD8^-^) or Treg (FOXP3^+^CD3^+^CD8^-^) phenotype, whereas only low numbers of Th1, Th17, and Tfh cells were observed. Similarly, Th2 was the most abundant Th subset in TLS, followed by Treg cells. In sharp contrast, Th1 was the most frequently detected Th subset in non-cancerous lung tissue from the same patients. A higher Th1:Th2 ratio in tumor stroma was found to be associated with increased numbers of intratumoral CD8^+^ T cells. The predominance of Th2 and Treg cells in both tumor stroma and tumor epithelium was consistent for all the 11 patients investigated. We conclude that human primary NSCLC tumors are Th2-skewed and contain numerous Treg cells. If human tumors are Th2-skewed, as our data in NSCLC suggest, reprogramming the type of immune response from a detrimental Th2 to a beneficial Th1 may be critical to increase the response rate of immunotherapy.

**Graphical Abstract:**
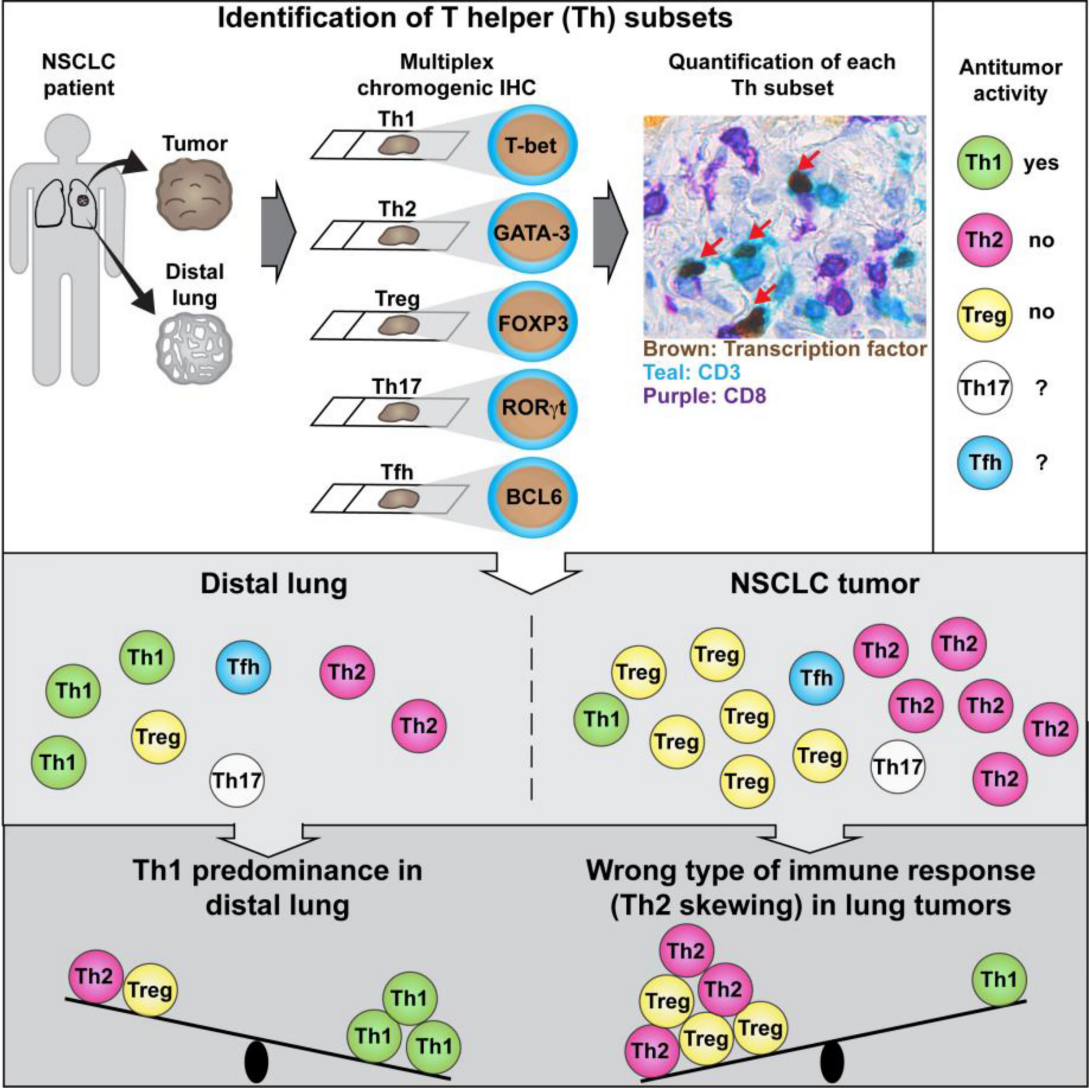
Tumor-infiltrating T helper (Th) lymphocytes may either suppress or promote tumor development depending on their activation phenotype. Frafjord et al. developed a method to quantify Th subsets in human tumors and revealed a wrong type of ongoing antitumor immune response (Th2 instead of Th1) in non-small cell lung cancer (NSCLC).

## Introduction

Lung cancer is the leading cause of cancer-related deaths worldwide, and non-small cell lung cancer (NSCLC) represents 85% of all lung cancer cases. Enhancement of T cell-mediated antitumor immunity using immune checkpoint inhibitors represented a breakthrough in the treatment of NSCLC, with a response rate typically around 20% and up to 45-48% in selected patient cohorts (1–3). However, complete responses are rare and many patients develop secondary resistance (4). Because lung carcinoma cells have a high mutation rate, they are expected to harbor many neoantigens and thereby be quite immunogenic (5). In fact, NSCLC tumors are being infiltrated by T cells (6), and high numbers of CD4^+^ T cells or CD8^+^ T cells in the stroma of NSCLC tumors have been reported to be associated with prolonged patient survival (7). The role of cytotoxic CD8^+^ T cells in killing cancer cells is well established and served as a basis for the development of immune checkpoint blockade immunotherapy. In contrast, CD4^+^ T cells have not received as much attention, although mouse studies have revealed the central role of tumor-specific CD4^+^ T cells in antitumor immunity (8–10). In humans, the importance of CD4^+^ T cells in preventing lung cancer is indirectly suggested by the observations that HIV-infected patients have a higher incidence of lung cancer, and lung cancer risk in HIV patients was shown to be associated with low CD4^+^ T cell counts (11–13). A high proportion of memory CD4^+^ T cells in the blood of patients with NSCLC has also been reported to be associated with clinical response to immune checkpoint inhibitors (14).

Deciphering the role of CD4^+^ T cells in cancer is complicated by the existence of several functional subsets such as T helper 1 (Th1) (15), Th2 (15), Th17 (16, 17), regulatory T (Treg) (18), and follicular helper T (Tfh) cells (19), which have very different and antagonizing properties. In fact, it has been proposed that some Th subsets may be tumor-suppressive while others may promote tumor development. Tumor-specific Th1 cells are known to provide essential help to CD8^+^ T cells to kill cancer cells, and have also been reported to suppress tumor development by inhibiting angiogenesis and by inducing the tumoricidal activity of macrophages (9, 10, 20). Treg cells are generally considered to be detrimental by dampening anticancer immunity and by contributing to the immunosuppressive microenvironment of tumors (21). A tumor-promoting role of Th2 cells has been proposed by several investigators. According to Bretscher’s Th2-skewing hypothesis, successful anti-cancer immunity is mediated by Th1 and CD8^+^ T cells, whereas a prevalent mechanism of tumor escape is the occurrence of a substantial Th2 component in the antitumor immune response (22, 23). A key concept behind this hypothesis is that different types of immune responses, such as Th1 and Th2, are known to suppress each other. The Th2-skewing hypothesis of tumor escape is based on mouse experiments but it has also received some support from human studies in renal cell carcinoma, melanoma, pancreatic carcinoma, and cutaneous T-cell lymphoma (24–26). Two related models by Mills and Mantovani proposed that tumor development is critically affected by the activation state of tumor-associated macrophages (M1 or M2), which is linked to the Th subset involved (Th1 or Th2, respectively) (27–29). Th2 cells were suggested to play a central role in the development of primary tumors and metastases by secreting cytokines such as IL-4 and IL-13 that induce a tumor-promoting M2 or M2-like phenotype of tumor associated macrophages (28, 30).

Several studies have shown that high densities of FOXP3^+^ Treg cells in lung tumors are associated with shorter patient survival (31–33). In contrast, very little data are currently available concerning other Th subsets in NSCLC tumors. The presence of T-bet^+^ Th1 cells in lung tumors has been reported (34). However, the single immunostaining that was performed using an anti-T-bet monoclonal antibody (mAb) could not distinguish T-bet^+^ Th1 cells from T-bet^+^ CD8^+^ T cells (34). Another study reported the poor survival of NSCLC patients who had a high density of IL-17-producing cells in tumor stroma. The cells were presumably Th17 cells, although their identity was not fully established (35). Flow cytometry analysis of NSCLC tumor tissue revealed the presence of IFN-γ^+^ Th1 and IL-17^+^ Th17 cells, but Th2 cells were not investigated (36). Thus, several Th subsets have been detected in NSCLC tumors, but the current data are fragmentary and the exact Th composition of human lung tumors remains unknown.

In this study, we established a multiplex chromogenic immunohistochemistry (IHC) assay that allowed us to quantify the five main CD4^+^ T cell subsets (Th1, Th2, Th17, Tfh and Treg cells), as well as CD8^+^ T cells, in tumor sections from 11 NSCLC patients. In tumor epithelium and tumor stroma, most CD4^+^ T cells identified had either a Th2 or Treg phenotype, while only low numbers of Th1, Th17 and Tfh cells were observed. Similarly, Th2 was the most abundant Th subset in peritumoral tertiary lymphoid structures (TLS), followed by Treg cells. In contrast, Th1 was the most frequently detected Th subset in non-cancerous lung tissue from the same patients. Our findings indicate a wrong type (Th2 instead of Th1) of ongoing antitumor immune response in human lung cancer, which may potentially explain why some NSCLC patients do not respond to immunostimulatory treatment with immune checkpoint inhibitors.

## Results

### A Multiplex Chromogenic IHC Assay for Detection of Th Subsets in Human Lung Tumors

**Figure 1:**
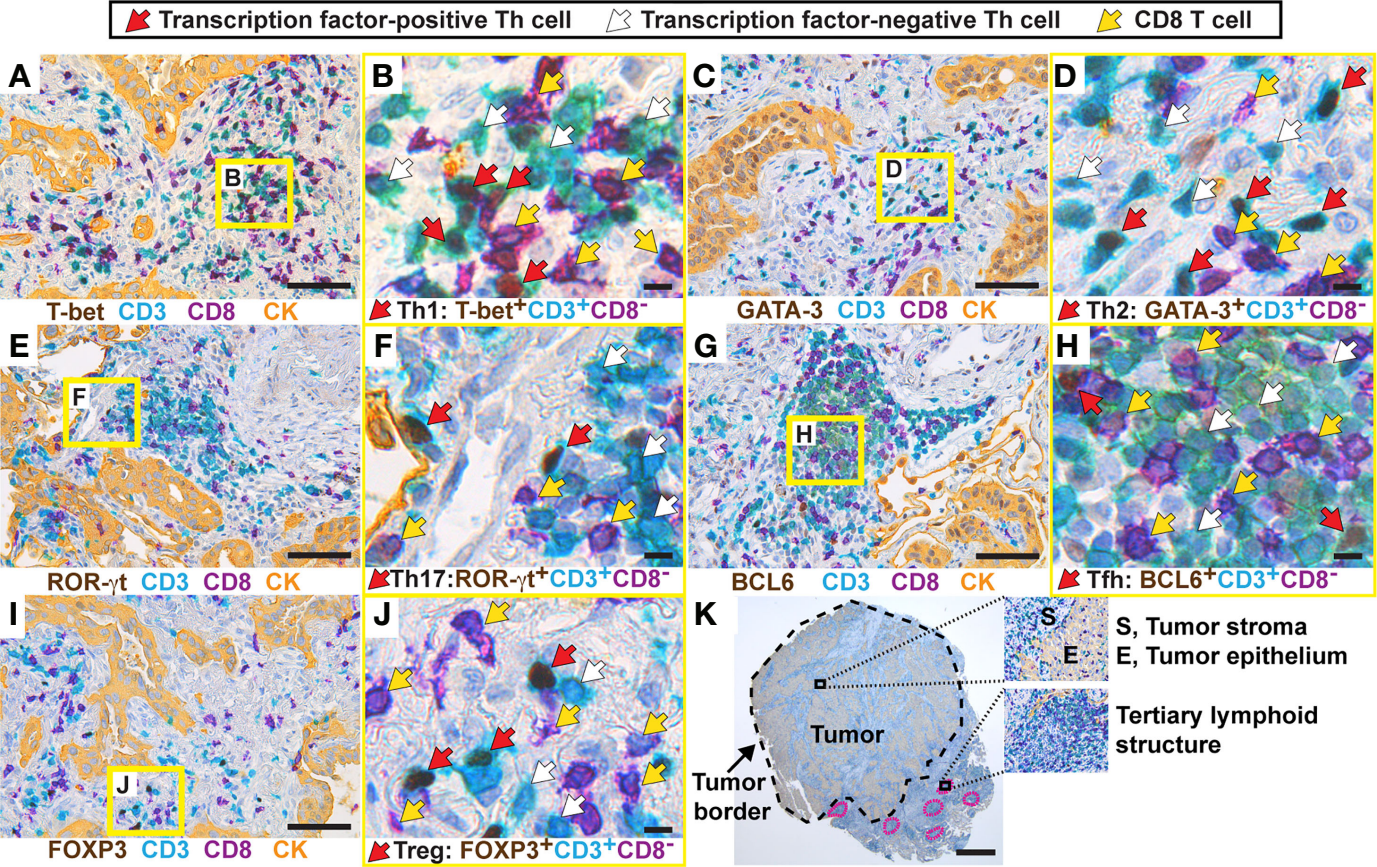
Establishment of a multiplex chromogenic IHC assay to detect five different Th subsets in human lung tumors. From each tumor (here, a lung adenocarcinoma), five 3 μm-thick serial sections were generated. T cells were immunostained with anti-CD3 (teal) and anti-CD8 (purple) mAbs, and all Th cells were identified as CD3^+^CD8^-^. Th subsets were detected using mAbs against subset-defining transcription factors (brown). Immunostaining of cytokeratins (CK) was used to visualize the tumor epithelium (yellow), and tumor stroma was defined as the cytokeratin-negative areas within the tumor border. Images containing higher than average densities of positive cells were selected for this figure. **(A)** Th1 cells were identified as T-bet^+^CD3^+^CD8^-^ cells, and the squared area indicated in yellow is shown in **(B)** with higher magnification. **(C, D)** Th2 cells were identified as GATA-3^+^CD3^+^CD8^-^. **(E, F)** Th17 cells were identified as ROR-γt^+^CD3^+^CD8^-^. **(G, H)** Tfh cells were identified as BCL6^+^CD3^+^CD8^-^. **(I, J)** Treg cells were identified as FOXP3^+^CD3^+^CD8^-^. **(K)** Image of a whole tumor and magnified areas illustrating three microanatomical regions that were analyzed in this study. The tumor border is marked with a black stippled line, and tertiary lymphoid structures are marked with a pink stippled line. In the high magnification images **(B, D, F, H, J)**, arrows indicate examples of positive/negative cells: red arrows point at transcription factor-positive Th cells (teal cells with brown nuclei), white arrows point at transcription factor-negative Th cells (teal cells with hematoxylin-stained purplish blue nuclei), and yellow arrows point at CD8^+^ T cells (purple). Original magnification: 400x **(A–J)**, 25x **(K)**. Scale bars: 50 μm **(A, C, E, G, I)**; 5 μm **(B, D, F, H, J)**; and 1 mm **(K)**.

The following immunostaining strategy was applied to detect the five major Th subsets (Th1, Th2, Th17, Tfh, and Treg) in serial sections from formalin-fixed, paraffin-embedded (FFPE) NSCLC tumors. All T cells and CD8^+^ T cells were identified using anti-CD3 (teal stain) and anti-CD8 (purple stain) mAbs, respectively. Th cells were identified as CD3^+^CD8^-^ cells (**Figure 1**). CD4 was not used as a marker for Th cells because human macrophages, which are frequent in tumors, express CD4. Importantly, we have previously shown using flow cytometry that the vast majority of T cells in NSCLC tumors were either CD3^+^CD4^+^ or CD3^+^CD8^+^, with very few CD3^+^ T cells being CD4^-^CD8^-^ double-negative (6). On each serial section, a Th subset was identified by combining the CD3^+^CD8^-^ staining with a mAb specific for a subset-defining transcription factor (brown stain): T-box expressed in T cells (T-bet) (37) for Th1 cells (**Figures 1A, B**); GATA-3 (38) for Th2 cells (**Figures 1C, D**); retinoic-acid-receptor-related orphan receptor γt (ROR)-γt (39) for Th17 cells (**Figures 1E, F**); B-cell lymphoma 6 (BCL6) (40, 41) for Tfh cells (**Figures 1G, H**); and forkhead box P3 (FOXP3) (18) for Treg cells (**Figures 1I, J**). In the high magnification images (**Figures 1B, D, F, H, J**), red arrows indicate transcription factor-positive Th cells (teal cells with brown nuclei), white arrows indicate transcription factor-negative Th cells (teal cells with hematoxylin-stained purplish blue nuclei), and yellow arrows point at CD8^+^ T cells (purple). Carcinoma cells were identified using a pan-cytokeratin mAb mixture and visualized with a yellow stain (**Figures 1A, C, E, G, I**). Within the tumor border, the yellow-stained areas were defined as tumor epithelium, whereas regions without yellow stain were defined as tumor stroma.

Four distinct microanatomical regions were analyzed in this study: tumor epithelium, tumor stroma, peritumoral tertiary lymphoid structures (TLS) (**Figure 1K**), as well as non-cancerous distal lung tissue that was sampled furthest away from the tumor in the resected lobe. To establish the multiplex IHC protocols, we used positive control tissues expected to contain the particular Th subset: kidney transplant rejection for Th1 cells (42); intestinal worm infection for Th2 cells (43); gut tissue with Crohn´s disease for Th17 cells (44); and tonsils for Tfh cells (19) and Treg cells (**Supplementary Figures 1, 2**). Both moderate and high signal intensities of brown nuclei were observed in the positive controls and in the NSCLC tumor tissue. Isotype- and concentration-matched negative control mAbs showed virtually no unspecific staining (**Supplementary Figures 3, 4**). Because of the low numbers of Th1 and Tfh positive cells in tumor tissue, these immunostainings were validated by comparing two different mAb clones for each transcription factor (T-bet and BCL6), and similar results were obtained (**Supplementary Figures 5, 6**). Thus, a four-color multiplex chromogenic IHC assay was successfully established for the identification of the five major Th subsets (Th1, Th2, Th17, Tfh and Treg) in human primary lung tumors.

### Th2 and Treg Cells Predominate in NSCLC Tumor Epithelium and Tumor Stroma

**Figure 2:**
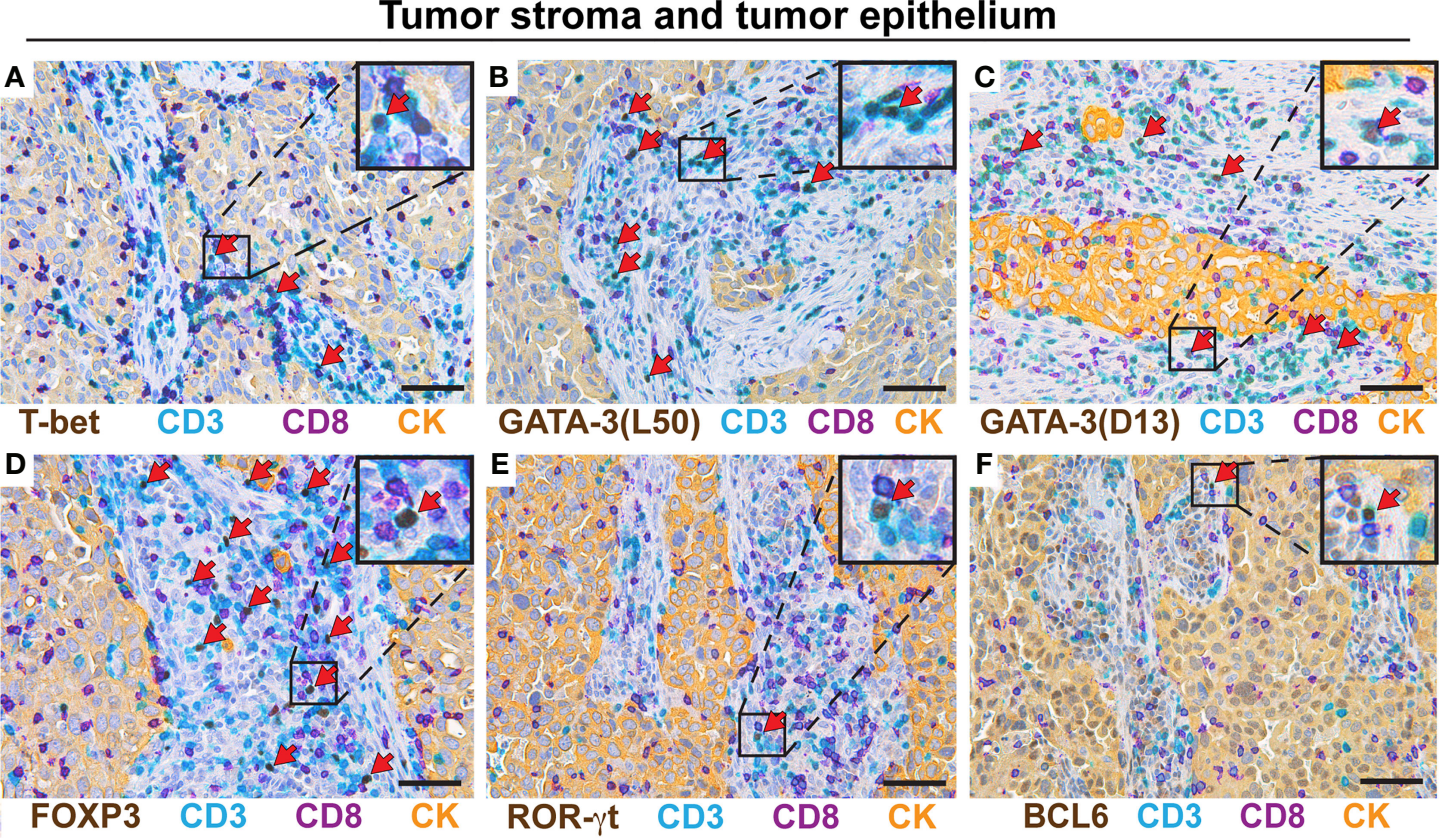
Th2 and Treg cells predominate in NSCLC tumor stroma and epithelium. Representative images of stained serial sections from a primary lung adenocarcinoma tumor. Boxed areas are enlarged and presented as corner insets. T cells were stained with anti-CD3 (teal) and anti-CD8 (purple) mAbs, and CD3^+^CD8^-^ cells were defined as Th cells. Th cells were identified using mAbs specific for subset-defining transcription factors (brown). The positive cells for each Th subset (teal cells with brown nuclei) are indicated by red arrows. Immunostaining of cytokeratins (CK) was used to visualize the tumor epithelium that contains the carcinoma cells (yellow), and tumor stroma was defined as cytokeratin-negative areas within the tumor border. **(A)** Th1 cells (T-bet^+^CD3^+^CD8^-^). **(B)** Th2 cells (GATA-3^+^CD3^+^CD8^-^) identified by the anti-GATA-3 mAb clone L50-816 designated L50. **(C)** Th2 cells (GATA-3^+^CD3^+^CD8^-^) identified by the anti-GATA-3 mAb clone D13C9 abbreviated D13. **(D)** Treg cells (FOXP3^+^CD3^+^CD8^-^). **(E)** Th17 cells (ROR-γt^+^CD3^+^CD8^-^). **(F)** Tfh cells (BCL6^+^CD3^+^CD8^-^). Original magnification, 400x. Scale bars, 50 μm.

Eleven patients with NSCLC were included in this study: six adenocarcinomas, four squamous cell carcinomas (SCC), and one adenosquamous carcinoma (ASC) (**Table 1**). The included patients had not received any treatment for NSCLC prior to surgery and sample collection. Serial sections from each tumor were immunostained using the established multiplex IHC protocols, and Th subsets were quantified manually. **Figure 2** illustrates the typical density and localization in lung tumors of Th1 cells (**Figure 2A**), Th2 cells (**Figures 2B, C**), Treg cells (**Figure 2D**), Th17 cells (**Figure 2E**), and Tfh cells (**Figure 2F**). Most Th cells were localized in the tumor stroma, with only a few Th cells infiltrating the tumor epithelium. NSCLC tumor stroma typically contained many Th2 and Treg cells, whereas only a few Th1, Th17 and Tfh cells were observed (**Figure 2**). Immunostaining with two different anti-GATA-3 mAb clones was compared, and high numbers of Th2 cells were consistently detected, although relatively fewer positive cells were obtained with clone D13C9 as compared to clone L50-816 (**Figures 2B, C**). We concluded that anti-GATA-3 mAb clone L50-816 was more sensitive and thereby more reliable for Th2 cell quantification.

**Table 1:** Characterization of the NSCLC patient cohort (n=11).

Age-year	Mean	66.8
	Range	42-83
Gender (%)	Male	5 (45)
	Female	6 (55)
Smoking status^*^ (%)	Smoker	7 (64)
	Former	3 (27)
	Never-smoker	1 (9)
Histology (%)	Adenocarcinoma	6 (55)
	Squamous cell carcinoma	4 (36)
	Adenosquamous carcinoma	1 (9)
pTNM stage (%)	IA	3 (27)
	IB	0 (0)
	IIA	0 (0)
	IIB	3 (27)
	IIIA	4 (36)
	IIIB	1 (9)
Procedure (%)	Lobectomy	6 (55)
	Sleeve lobectomy	1 (9)
	Pneumectomy	3 (27)
	Bilobectomy	1 (9)
Tumor location (%)	Right upper lobe	3 (27)
	Right middle lobe	1 (9)
	Right lower lobe	2 (18)
	Left upper lobe	3 (27)
	Left lower lobe	2 (18)
Concomitant disease (%)	COPD^**^	4 (36)
	Heart diseases	4 (36)

*Smokers refer to patients who were actively smoking at the time of the operation and those who quitted smoking <1 year before diagnosis. Former smokers refer to patients who had stopped smoking ≥1 year prior to diagnosis.

**COPD, chronic obstructive pulmonary disease.

**Figure 3:**
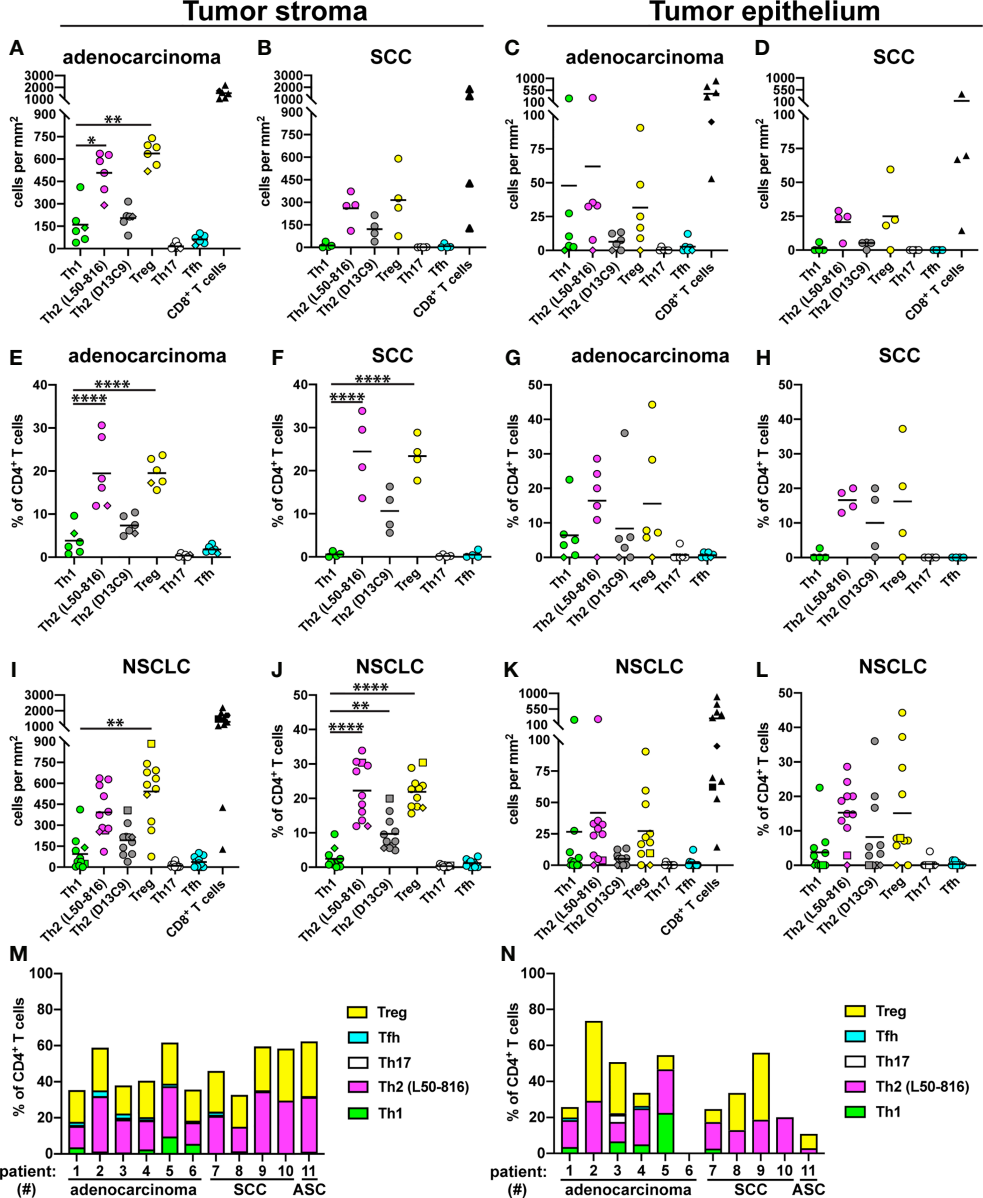
Quantification of Th subsets and CD8^+^ T cells in tumor stroma and epithelium. Tumors from 11 NSCLC patients [6 adenocarcinoma, 4 squamous cell carcinoma (SCC), and 1 adenosquamous carcinoma (ASC)] were immunostained as shown in the previous figures, and cells were quantified. Each symbol in the graphs represents one patient and black lines indicate mean values. The rhombus symbol (♦) indicates the only never-smoker patient. **(A)** Cell density (number of cells per mm^2^) in the stroma of adenocarcinoma tumors, or **(B)** SCC tumors. **(C)** Cell density in the tumor epithelium of adenocarcinoma, or **(D)** SCC tumors. **(E)** Percentage of each Th cell subset (of all CD3^+^CD8^-^ Th cells) in the stroma of adenocarcinoma tumors, or **(F)** SCC tumors. **(G)** Percentage of each Th cell subset in the tumor epithelium of adenocarcinoma, or **(H)** SCC tumors. **(I–L)** Pooled data for all NSCLC patients. The square symbol indicates the adenosquamous carcinoma patient. **(I)** Cell density data in tumor stroma. **(J)** Th percentages in tumor stroma. **(K)** Cell density data in tumor epithelium. **(L)** Th percentages in tumor epithelium. **(M)** Th subset composition (as percent of all CD3^+^CD8^-^ Th cells) for each patient in tumor stroma, or **(N)** in tumor epithelium. L50-816 and D13C9 indicate two different anti-GATA-3 mAb clones. Statistical analysis was performed using one-way ANOVA with Bonferroni´s *post-hoc* test. *p<0.05, **p<0.01, ****p<0.0001.

Cell quantification in tumor stroma for adenocarcinoma patients revealed high densities of Treg cells (mean: 640 cells/mm^2^) and Th2 cells (500 cells/mm^2^) and relatively low levels of Th1 cells (160 cells/mm^2^) (**Figure 3A**). A similar pattern was observed in the SCC tumor stroma (**Figure 3B**), and in the tumor epithelium of both adenocarcinoma and SCC (**Figures 3C, D**), although the differences did not reach statistical significance. When calculating the relative frequency of each Th subset, expressed as percentage of all CD3^+^CD8^-^ Th cells, Treg cells and Th2 cells were found to each represent approximately 20-25% of all CD4^+^ T cells, both in adenocarcinoma and SCC (**Figures 3E, F**). In contrast, Th1 cells made up only 2-3% in average of the CD4^+^ T cell population, and the proportions of Th17 and Tfh cells were even lower (**Figures 3E, F**). In the tumor epithelium, there was a similar trend toward Th2 and Treg cell predominance (**Figures 3G, H**). When considering the tumor stroma data for all NSCLC patients investigated, the density of Treg cells and the percentages of both Treg and Th2 cells were significantly higher compared to Th1 (**Figures 3I, J**). The mean percentages (of all CD3^+^CD8^-^ cells) in tumor stroma were: Th2 (22%), Treg (22%), Th1 (2.4%), Tfh (1.2%), and Th17 (0.3%) (**Figure 3J**). Tumor epithelium data for all patients did not show statistically significant differences, although the frequencies of Th2 and Treg cells generally seemed to be highest (**Figures 3K, L**). In all analyses, Th17 cells and Tfh cells were found to be rare in both tumor stroma and tumor epithelium (**Figure 3**). Bar graph presentation of the Th subset percentages for individual patients reveals a strong and consistent predominance of Th2 and Treg cells in both tumor stroma and tumor epithelium in NSCLC (**Figures 3M, N**). We could thus conclude that NSCLC tumors are Th2 skewed and contain many Treg cells.

### Th2 Cells Predominate in Tertiary Lymphoid Structures

**Figure 4:**
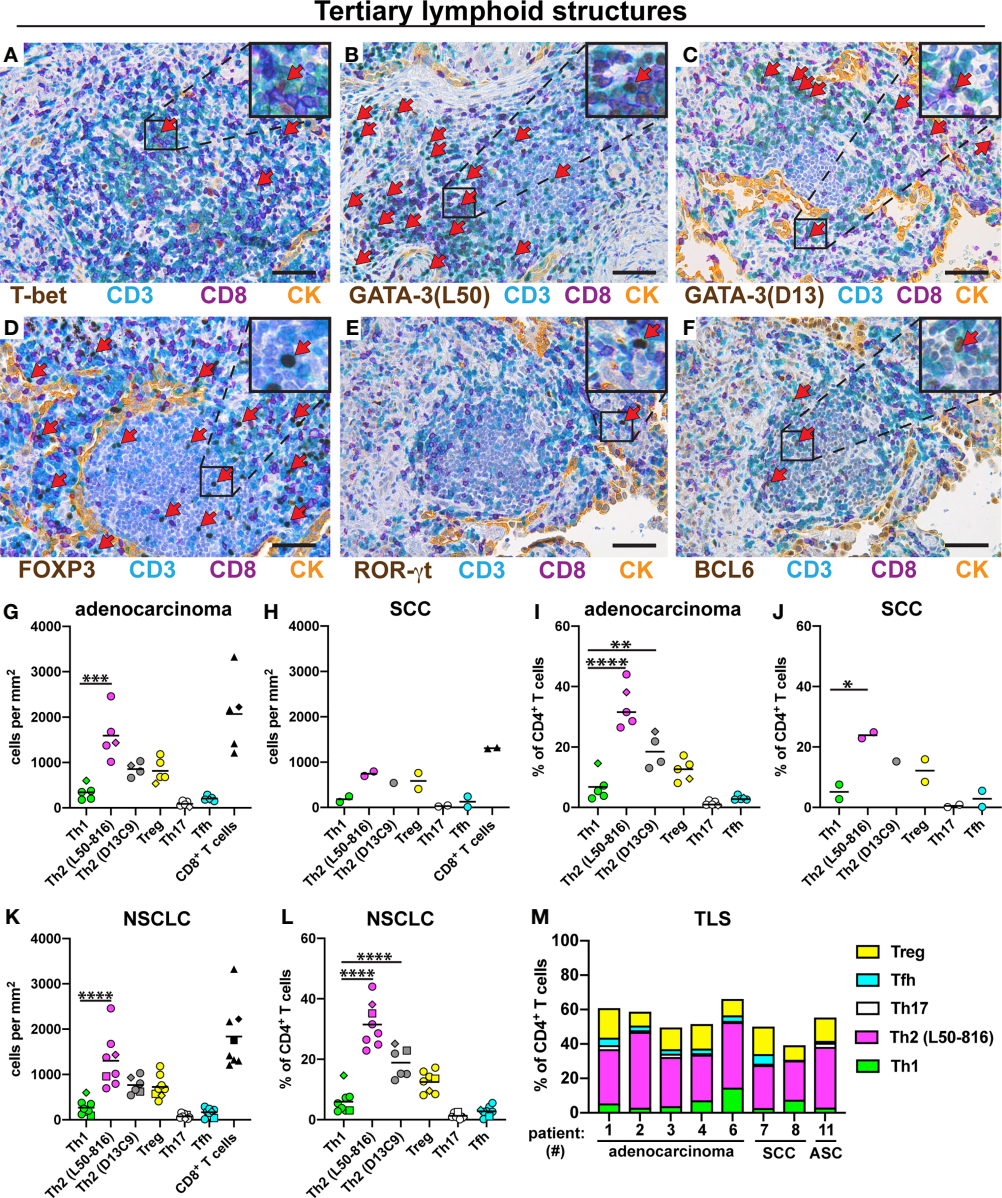
Quantification of Th subsets and CD8^+^ T cells in tertiary lymphoid structures (TLS). Tumor sections from 8 NSCLC patients [5 adenocarcinoma, 2 squamous cell carcinoma (SCC), and 1 adenosquamous carcinoma (ASC)] were immunostained and cells were quantified in TLS located at the tumor periphery. **(A–F)** Representative images of stained TLS. Boxed areas are enlarged and presented as corner insets. Red arrows indicate positively stained cells for each Th subset. **(A)** Th1 cells (T-bet^+^CD3^+^CD8^-^). **(B, C)** Th2 cells (GATA-3^+^CD3^+^CD8^-^) detected by mAb clones L50-816 and D13C9, respectively. **(D)** Treg cells (FOXP3^+^CD3^+^CD8^-^). **(E)** Th17 cells (ROR-γt^+^CD3^+^CD8^-^). **(F)** Tfh cells (BCL6^+^CD3^+^CD8^-^). **(G–M)** Quantitative data for T cell subsets in TLS. **(G)** Number of cells per mm^2^ in TLS of adenocarcinoma, or **(H)** SCC. **(I)** Percentage of each Th subset (of all CD3^+^CD8^-^ Th cells) in TLS of adenocarcinoma, or **(J)** SCC. **(K)** Pooled cell density data in TLS for all NSCLC patients. **(L)** Pooled NSCLC data for Th percentages in TLS. **(M)** Th subset composition in TLS (as percent of all CD3^+^CD8^-^ cells) for each patient analyzed. Each symbol in the graphs represents one patient and black lines indicate mean values. The square symbol indicates the adenosquamous carcinoma patient. The rhombus symbol (♦) indicates the never-smoker patient. Statistical analysis was performed using one-way ANOVA with Bonferroni´s *post-hoc* test. *p<0.05, **p<0.01, ***<0.001, ****p<0.0001. CK, cytokeratin. Original magnification, 400x. Scale bars, 50 μm.

TLS are lymph node-like structures that are located at the periphery of NSCLC tumors and have been reported to contain mostly T and B cells, as well as some dendritic cells (34). Th cells were quantified in TLS from eight patients with NSCLC. The following five Th subsets were found in TLS: Th1 cells (**Figure 4A**), Th2 cells (**Figures 4B, C**), Treg cells (**Figure 4D**), Th17 cells (**Figure 4E**), and Tfh cells (**Figure 4F**). The density of Th2 cells (average: 1600 cells/mm^2^) was significantly higher than that of Th1 cells (average: 340 cells/mm^2^) in TLS for adenocarcinoma (**Figure 4G**). A similar trend was observed for SCC (**Figure 4H**). When expressed as percentage of all CD4^+^ T cells, Th2 was the most frequent Th subset in TLS in both adenocarcinoma and SCC (**Figures 4I, J**). When pooling all NSCLC data, the frequency of Th2 cells (in density or percentage) was found to be significantly higher than that of Th1 cells (**Figures 4K, L**). The mean percentages of each Th subset among all CD3^+^CD8^-^ cells in TLS were: Th2 (32%), Treg (13%), Th1 (5.9%), Tfh (2.8%), and Th17 (1.3%). Bar graph presentation of Th subset percentages for each patient shows a strong Th2 predominance in TLS, and the presence of a smaller, but consistent Treg population, while other Th subsets were infrequent (**Figure 4M**).

### Th1 Is the Most Frequently Detected Th Subset in Distal Lung

**Figure 5:**
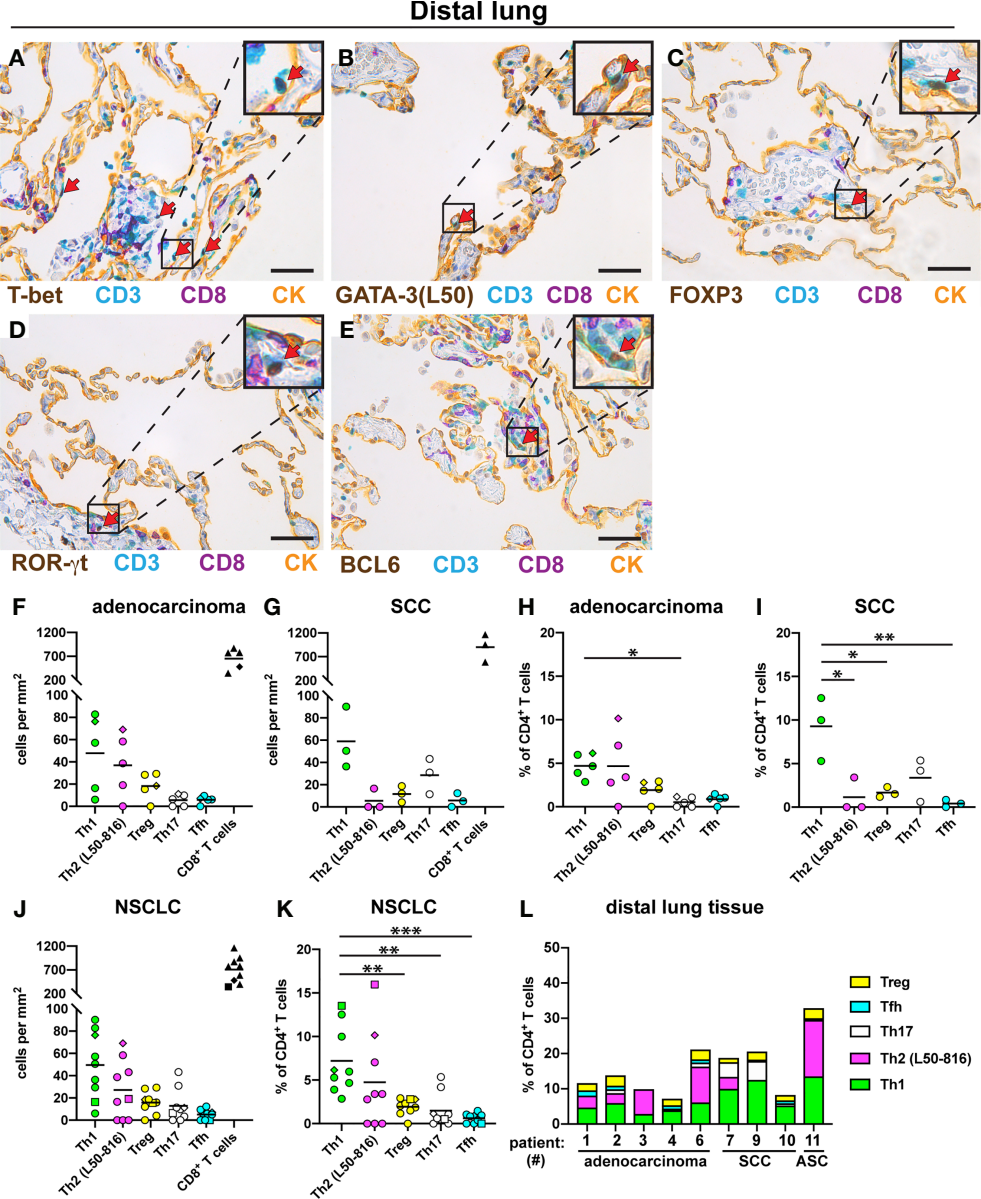
Quantification of Th cell subsets and CD8^+^ T cells in distal lung. Non-cancerous distal lung tissue was sampled furthest away from the tumor in the resected lobe from 9 NSCLC patients [5 adenocarcinoma, 3 squamous cell carcinoma (SCC), and 1 adenosquamous carcinoma (ASC)]. Serial sections were immunostained and cells were quantified. **(A–E)** Representative images of stained Th cells in distal lung. A pan-cytokeratin (CK) stain was used to visualize the alveolar epithelial cells (yellow). Boxed areas are enlarged and shown as corner insets. Positive cells for each Th subset are indicated by red arrows. **(A)** Th1 cells (T-bet^+^CD3^+^CD8^-^). **(B)** Th2 cells (GATA-3^+^CD3^+^CD8^-^) identified with anti-GATA-3 mAb clone L50-816 (L50). **(C)** Treg cells (FOXP3^+^CD3^+^CD8^-^). **(D)** Th17 cells (ROR-γt^+^CD3^+^CD8^-^). **(E)** Tfh cells (BCL6^+^CD3^+^CD8^-^). **(F–L)** Quantitative data for T cell subsets in distal lung. **(F)** Number of cells per mm^2^ in distal lung of adenocarcinoma, or **(G)** SCC. **(H)** Percentage of each Th subset (of all CD3^+^CD8^-^ Th cells) in distal lung of adenocarcinoma, or **(I)** SCC. **(J)** Pooled cell density data in distal lung for all NSCLC patients. **(K)** Pooled NSCLC data for Th percentages in distal lung. **(L)** Th subset composition in distal lung (as percent of all CD3^+^CD8^-^ cells) for each patient. Each symbol in the graphs represents one patient and black lines indicate mean values. The square symbol indicates the adenosquamous carcinoma patient. The rhombus symbol indicates the never-smoker patient. Statistical analysis was performed using one-way ANOVA with Bonferroni´s *post-hoc* test. *p<0.05, **p<0.01, ***<0.001. Original magnification, 400x. Scale bars, 50 μm.

For comparison, we examined the Th composition in non-cancerous distal lung tissue from the same NSCLC patients. All five Th subsets were found: Th1 cells, Th2 cells, Treg cells, Th17 cells, and Tfh cells (**Figures 5A–E**). In sharp contrast to the tumor data, Th1 cells were relatively frequent in distal lung both in density and in percentage of all CD3^+^CD8^-^ Th cells (**Figures 5F–K**). When analyzing the data for all NSCLC patients, the percentage of Th1 cells (mean: 7.2%) in distal lung was found to be somewhat higher than that of Th2 cells (4.8%) and significantly higher than the percentage of Treg cells (1.9%), Th17 cells (1.5%) and Tfh cells (0.6%) (**Figure 5K**). Bar graph presentation revealed that the Th composition in distal lung was quite heterogeneous among the NSCLC patients, although Th1 cells were consistently well represented (**Figure 5L**). Thus, in contrast to the situation observed in tumor stroma and tumor epithelium, Th1 was the most frequently detected Th subset in non-cancerous distal lung from the same NSCLC patients.

### The Th Composition Varies Markedly in Different Microanatomical Regions

**Figure 6:**
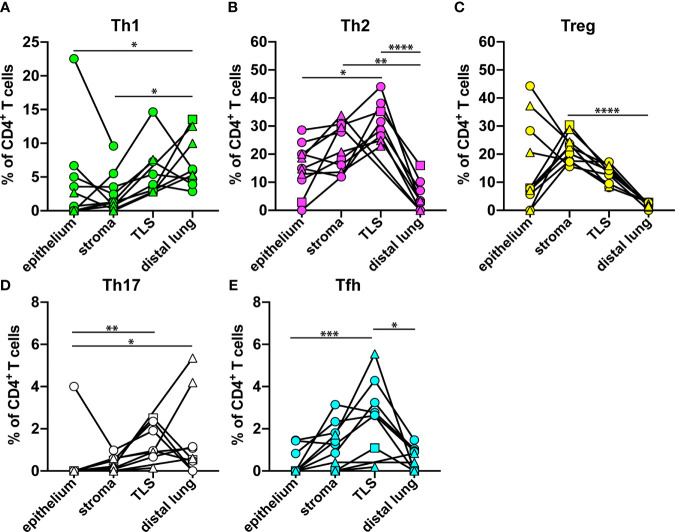
Relative frequency of each Th cell subset in different microanatomical regions. The data from **Figures 3**–**5** were used to compare the percentage of each Th subset (of all CD3^+^CD8^-^ Th cells) in tumor epithelium (n=11), tumor stroma (n=11), tertiary lymphoid structure (TLS, n=8), and distal lung (n=9) of NSCLC patients. **(A)** Th1 cells (T-bet^+^CD3^+^CD8^-^). **(B)** Th2 cells (GATA3^+^CD3^+^CD8^-^) detected by mAb clone L50-816. **(C)** Treg cells (FOXP3^+^CD3^+^CD8^-^). **(D)** Th17 cells (ROR-γt^+^CD3^+^CD8^-^). **(E)** Tfh cells (BCL6^+^CD3^+^CD8^-^). Each symbol in the graphs represents one patient. Squamous cell carcinoma patients are indicated by triangles, and adenocarcinoma patients by circles. The square symbol indicates the adenosquamous carcinoma patient. Statistical calculations were performed with non-parametric Kruskal-Wallis analysis and Dunn´s *post-hoc* test. *p<0.05, **p<0.01, ***p<0.001, ****p<0.0001.

The relative frequency of each Th subset, as percent of all CD3^+^CD8^-^ Th cells, in the four microanatomical regions investigated, was compared for all NSCLC patients (**Figure 6**). The percentage of Th1 cells was significantly decreased in tumor epithelium and tumor stroma as compared to distal lung (**Figure 6A**). Strikingly, the percentage of Th2 cells was significantly increased in tumor stroma and in TLS compared to distal lung (**Figure 6B**). The percentage of Treg cells was found to be significantly higher in tumor stroma compared to distal lung (**Figure 6C**). Th17 cells were more frequent in TLS and distal lung than in the tumor epithelium (**Figure 6D**). Finally, the percentage of Tfh cells was higher in TLS than in tumor epithelium and distal lung (**Figure 6E**), which is in accordance with the predicted role of Tfh cells in helping B cells to produce antibodies in TLS (19). Thus, the location appears to have a strong impact on the Th composition: Th2 and Treg cells dominate the tumor microenvironment, including tumor-associated TLS, whereas Th1 cells are relatively frequent in non-cancerous distal lung tissue.

### Immunostaining of CXCR3 and CCR4 Confirms the Low Th1:Th2 Ratio in Tumor Stroma and TLS

**Figure 7:**
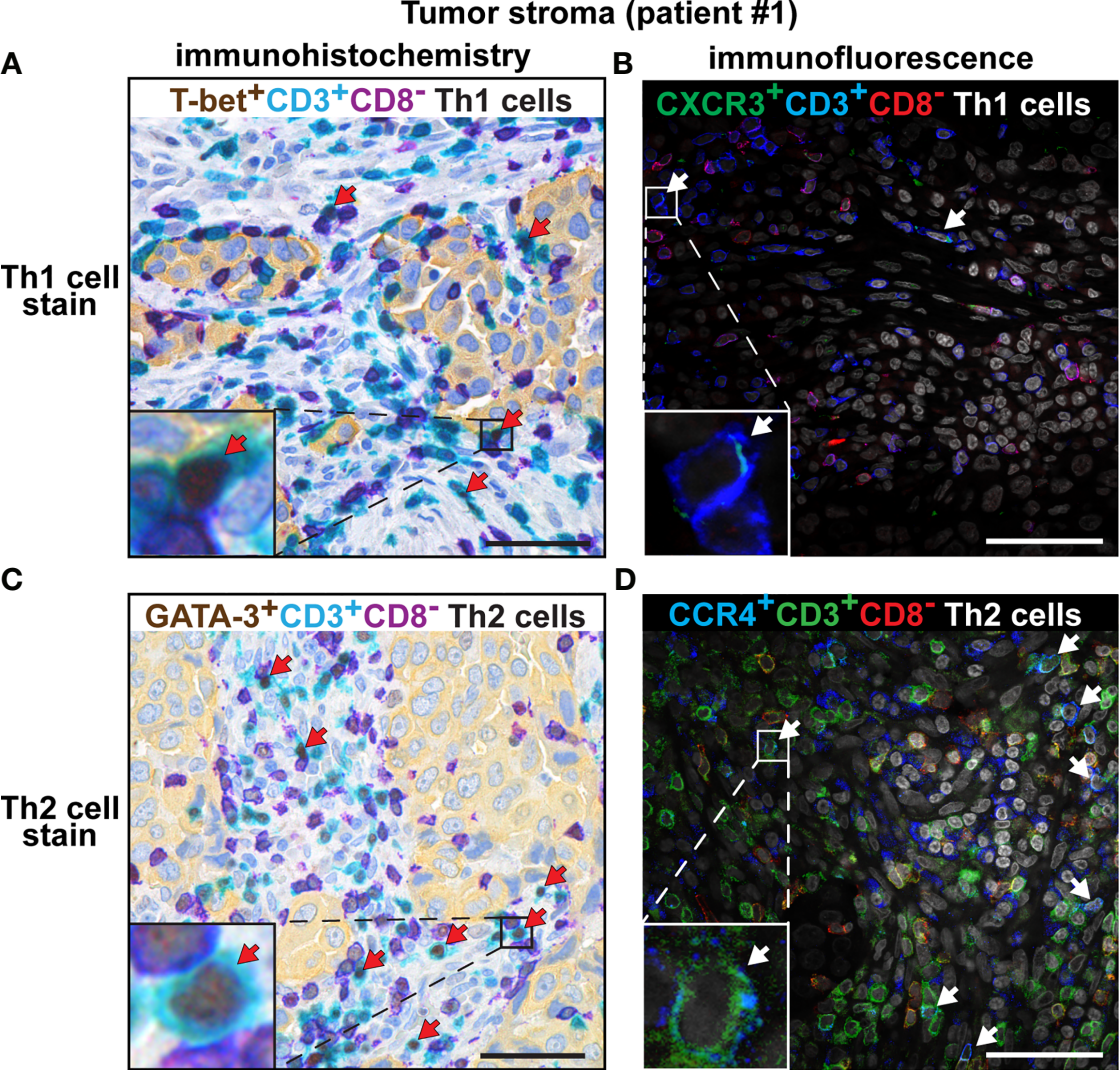
Comparison of the number of intratumoral Th1 and Th2 cells detected by chromogenic IHC *vs.* immunofluorescence. Representative images of immunostained tumor sections from one patient with lung adenocarcinoma. Boxed areas are enlarged and presented as corner insets. The positive cells for each Th subset in tumor stroma are indicated by red or white arrows. **(A)** Th1 cells (T-bet^+^CD3^+^CD8^-^) detected by chromogenic IHC. **(B)** Th1 cells (CXCR3^+^CD3^+^CD8^-^) detected by immunofluorescence. **(C)** Th2 cells (GATA-3^+^CD3^+^CD8^-^) detected by chromogenic IHC, using the anti-GATA-3 mAb clone L50-816. **(D)** Th2 cells (CCR4^+^CD3^+^CD8^-^) detected by immunofluorescence. Similar results were obtained from all three investigated NSCLC patients. Original magnification, 600x. Scale bars, 50 μm.

Th subset quantification by multiplex chromogenic IHC revealed a high density of Th2 cells and a low density of Th1 cells in tumor stroma, consistent with a Th2-skewing of the immune landscape. To confirm these findings using a different method, we performed immunofluorescence staining of three cases of adenocarcinoma with antibodies against chemokine receptors known to be associated with Th1 and Th2 cells, respectively. Th1 cells have been reported to express the chemokine receptor CXCR3, whereas the chemokine receptor CCR4 is expressed by Th2 cells (45). CD4^+^ T cells were identified based on the combination of positive CD3 expression and negative CD8 expression. Many more Th2 cells (CCR4^+^CD3^+^CD8^-^) than Th1 cells (CXCR3^+^CD3^+^CD8^-^) were detected in tumor stroma (**Supplementary Figure 7**) and in TLS (**Supplementary Figure 8**) by immunofluorescence. Side by side comparison of the data obtained by chromogenic IHC versus immunofluorescence revealed a consistent staining pattern with high numbers of Th2 cells and low numbers of Th1 cells in tumor stroma (**Figure 7** and **Supplementary Figure 9**) and in TLS (**Supplementary Figure 10**). The isotype-matched control mAbs for CXCR3 and CCR4 showed virtually no unspecific staining (**Supplementary Figure 11**). Thus, immunofluorescence staining of the chemokine receptors CXCR3 and CCR4 confirms the low Th1:Th2 ratio and thereby the Th2-skewing of the immune landscape in tumor stroma and TLS.

### There Is a Positive Correlation Between the Th1:Th2 Ratios in Tumor Stroma and the Number of Tumor-Infiltrating CD8^+^ T Cells

**Figure 8:**
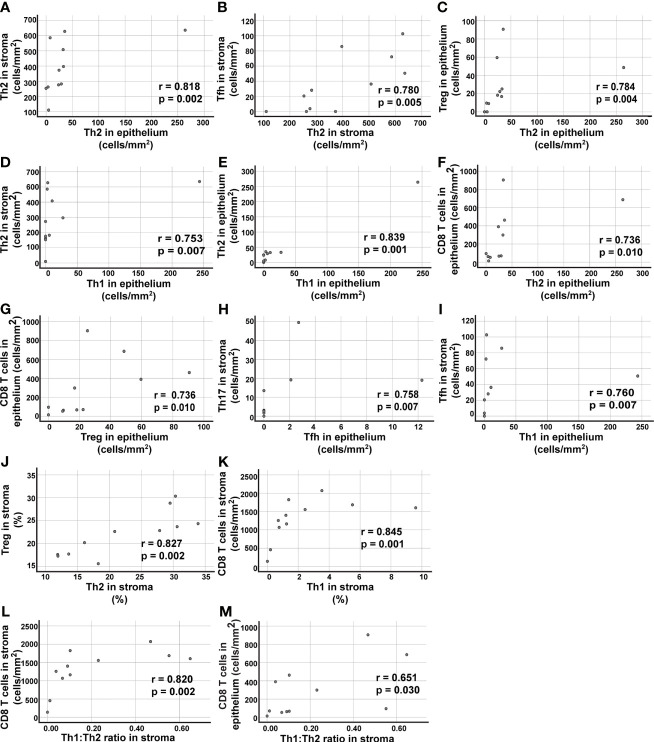
Correlation analysis of T cell data in tumor epithelium and tumor stroma. Spearman´s rho was used to analyze the data presented in **Figure 3** and identify correlations. **(A–I)** Nine of the tested combinations of T cell density data (cells/mm^2^) resulted in p values ≤0.01 and positive correlation r>0.7. For a complete list of analyzed combinations, see **Supplementary Table 2**. **(J, K)** Two combinations of T cell subsets showed positive correlation with p ≤ 0.01 and r>0.7 when percent numbers for the Th cell subsets were used in the correlation analysis (for a complete list of analyzed combinations, see **Supplementary Table 3**). **(L)** The ratio of Th1:Th2 cells in stroma showed a positive correlation with the density (cells/mm^2^) of CD8^+^ T cells in tumor stroma, and **(M)** in tumor epithelium. The Th2 data included in the correlation analysis was from the immunostainings using the anti-GATA-3 clone L50-816. Each symbol represents data from one NSCLC patient.

Spearman’s Rank Correlation Coefficient was used to analyze the data (**Figure 8** and **Supplementary Tables 1–6**). The strongest positive correlations detected, as defined by Spearman ρ > 0.7 and *p* ≤ 0.01, are reported in **Figure 8**. There was a strong correlation between the density of Th2 cells in tumor stroma and tumor epithelium (**Figure 8A**). The density of Th2 cells was found to be associated with the density of several other T cell subsets such as Tfh and Treg cells (**Figures 8B, C**). More surprisingly, the density of Th1 cells in tumor epithelium (which arguably was very low) was positively associated with Th2 cell density in tumor stroma and epithelium (**Figures 8D, E**). In tumor epithelium, there was also a surprising positive correlation between densities of CD8^+^ T cells and numbers of either Th2 or Treg cells (**Figures 8F, G**). Notably, the relative frequency of Th2 and Treg cells (expressed as percent of all CD4^+^ T cells) showed a strong correlation in tumor stroma (**Figure 8J**). The percentage of Th1 cells in tumor stroma was associated with the density of CD8^+^ T cells, consistent with the role of Th1 cells in recruiting CD8^+^ T cells to the tumor (**Figure 8K**). In support of this model, a higher Th1:Th2 ratio in tumor stroma was found to be associated with a higher density of CD8^+^ T cells, in both tumor stroma and tumor epithelium (**Figures 8L, M**). In summary, there was a strong correlation between the percentages of Th2 and Treg cells in tumor stroma. There were also strong correlations between the percentage of Th1 cells, as well as the Th1:Th2 ratios, in tumor stroma, and the number of tumor-infiltrating CD8^+^ T cells.

## Discussion

We report the establishment of a multiplex chromogenic IHC assay that allows the identification of the five main CD4^+^ T cell subsets (Th1, Th2, Th17, Tfh, and Treg cells) in human tumors. Although IHC is certainly not a new technology, a simple and reliable protocol to identify Th subsets on tumor sections was lacking, essentially because of the following technical reasons. The protein expression levels of the canonical cytokines and ‘master regulator’ transcription factors that define each Th subset tend to be relatively low, which is suboptimal for immunostaining. Most IHC studies use one single mAb per slide, which is insufficient to identify Th cells, because the expression of the subset-defining transcription factors (and Th cytokines) is not restricted to CD4^+^ T cells. Another complication is that the Th marker CD4 is also expressed by human macrophages. Therefore, we had to establish and validate combinations of three mAbs (against CD3, CD8, and transcription factors) to identify Th subsets in lung tumor sections. A pan-cytokeratin stain was also included to visualize the cancer cells. This new method should be very useful to investigate and clarify the Th composition in human tumors of potentially any cancer type.

We provide here the first detailed analysis of Th subsets in human NSCLC. We found that most CD4^+^ T cells in tumor epithelium and tumor stroma had either a Th2 or Treg phenotype, whereas only low numbers of Th1, Th17 and Tfh cells were observed. Similarly, Th2 was the most abundant Th subset in TLS, followed by Treg cells. In contrast, Th1 was the most frequently detected Th subset in non-cancerous lung tissue from the same NSCLC patients. We could thus conclude that human NSCLC tumors are Th2-skewed and contain numerous Treg cells.

A limitation of the current study is that the patient cohort was relatively small (n=11) and clinically rather heterogeneous, in terms of cancer histological subtypes, clinical stages, and smoking status. However, the predominant Th2/Treg pattern was very clear and consistent among all the eleven patients investigated (see **Figures 3M, N**). Because patient inclusion was solely based on lung cancer diagnosis, the finding that eleven out of eleven tumors investigated were Th2-skewed is statistically a strong indicator that it represents the predominant pattern in NSCLC. Notably, a Th2-skewing was observed for the two main subtypes of NSCLC: adenocarcinoma (6 out of 6) and squamous cell carcinoma (4 out of 4), as well as for the single adenosquamous carcinoma patient investigated. Future studies with larger patient cohorts and taking advantage of the new method will be important to confirm our findings and potentially identify subgroups of patients with a different Th composition (such as a Th1 predominance). Larger studies may also reveal correlations between the intratumoral Th subset composition and patient prognosis or response to immunotherapy.

Th cells have the central function of regulating immune responses which they do mostly by secreting cytokines that control the activity of other effector immune cells. There are three main types of immune responses (type 1, 2, and 3) with distinct effector functions that are tailored to respond to different categories of pathogens (46). Each type of immune response is driven by a dedicated Th subset. Th1 cells govern type 1 immunity that is protective against intracellular microbes, and is also considered to be the most appropriate response to fight cancer (9, 10, 20). Th2 cells orchestrate type 2 immunity against parasites and venoms. Th17 cells control type 3 immunity to eliminate extracellular bacteria and fungi (46). Tfh cells participate in both type 1 and type 2 immune responses (47), while Treg cells prevent immunopathology by suppressing detrimental immune responses of any type (48). Importantly, different Th subsets suppress each other by secreting cytokines. For example, IFN-γ produced by Th1 cells inhibits the differentiation of Th2 cells, whereas IL-4 that is secreted by Th2 cells suppresses the generation of Th1 cells (49, 50). IL-4 also inhibits IFN-γ production by effector Th1 cells (51). Therefore, our data strongly suggest that the type of ongoing immune response in NSCLC tumors is not only qualitatively inappropriate (Th2 instead of Th1), but also detrimental because Th2 (and Treg) cells may inhibit the anti-cancer activity of the few Th1 cells by secreting Th2-associated cytokines such as IL-4 and IL-10.

To identify Th2 cells, we used GATA-3 which is the master regulator of Th2 cell differentiation, and which is both necessary and sufficient for gene expression of the canonical Th2 cytokines IL-4, IL-5, and IL-13 (38, 52). GATA-3 has also been reported to inhibit Th1 cell differentiation (53, 54). Our data showing a predominance of GATA-3^+^ Th2 cells, and low numbers of T-bet^+^ Th1 cells, in NSCLC tumors, are in accordance with a previous report in which cytokines were quantified by ELISA in tumor tissue homogenates (55). It was shown that NSCLC tumors contain very little IFN-γ (that is associated with Th1), and significantly more Th2 cytokines (IL-4 and IL-5) than does normal lung tissue (55). In contrast to our findings, Kargl et al. used flow cytometry and reported that 40% of all CD4^+^ T cells in NSCLC tumors were IFN-γ producing Th1 cells (36). This discrepancy with our data may be due to technical issues. Flow cytometry analysis of intracellular cytokines usually requires *in vitro* stimulation of the cells which may affect the Th cell phenotype. Kargl et al. also used a modified flow cytometry protocol with overnight cell incubation with antibodies, instead of the usual 20-30 minutes, which could have led to increased non-specific staining (36). Yet, it will be important in future studies to confirm the predominance of Th2 and Treg cells in NSCLC tumors using other methods such as single cell RNASeq or imaging mass cytometry.

FOXP3, the master regulator of Treg cell differentiation (18), was used to identify Treg cells in tumor sections. In contrast to effector CD4^+^ T cells (Th1, Th2, Th17, and Tfh) that activate other immune cells, Treg cells are inherently suppressive (48), and considered to have a detrimental role in cancer by suppressing the antitumor immune response. Several studies have shown that high densities of FOXP3^+^ Treg cells in lung tumors were associated with shorter patient survival (31–33). Although FOXP3 is widely used as a universal Treg marker, it should be noted that FOXP3 expression is not restricted to Treg cells (56). Most human CD4^+^ and CD8^+^ T cells have been reported to upregulate FOXP3 upon activation (48, 57, 58). FOXP3 expression is considered to be transient in activated effector T cells and more stable in Treg cells. However, it implies that the FOXP3^+^ T cells that were detected in NSCLC tumor sections may either represent *bona fide* Treg cells or recently activated effector Th cells (Th1, Th2, Th17 or Tfh).

TLS are lymph node-like structures that are created at sites of persistent inflammatory disorders and around tumors such as in NSCLC (34, 59). TLS possess both T-cell areas and B-cell follicles (34, 59). In the TLS associated with NSCLC tumors, we found that Th2 was the most abundant Th subset, followed by Treg cells. In line with our findings, it has been previously reported that TLS of human lung tumors contained many CCR4^+^CD3^+^ T cells [CCR4 being preferentially expressed by Th2 cells (45)] and relatively high gene expression levels of the Th2-associated chemokines CCL17 and CCL22 (59). Although limited in numbers, we could clearly detect Tfh cells in TLS, which is consistent with a key role of Tfh cells in helping B cells to produce antibodies in secondary, and presumably tertiary, lymphoid organs. The simultaneous presence of Th2 and Tfh cells in TLS is reminiscent of the description of a type 2 immune response that was observed in the draining lymph nodes of mice experimentally infected by parasites (47, 60). According to a model proposed by Reinhardt et al, IL-4-competent cells can adopt either a Tfh or canonical Th2 effector cell fate in the lymph node depending on whether or not they encounter antigen presented by B cells (47). Therefore, the observed Th composition of TLS, with high numbers of Th2 and some Tfh cells, is consistent with an ongoing type 2 immune response in NSCLC tumors.

Collectively, our data provide support to Bretscher’s Th2-skewing hypothesis of tumor escape (22, 23). A wrong type of ongoing antitumor immune response (Th2 instead of Th1) may potentially explain why many NSCLC patients do not respond to immune checkpoint inhibitors, because enhancing an ongoing inappropriate type of immune response is unlikely to help the patient. If human tumors are indeed Th2-skewed, as our data in NSCLC suggest, it may be critical to reprogram the type of immune response from Th2 to Th1 to eradicate cancer. Preclinical studies suggest that intratumoral injections of viral vector encoding the Th1-polarizing cytokines IL-12 and IFN-γ may represent one approach to reach this goal (61, 62). Novel therapeutic strategies focusing on the quality of the antitumor immune response (Th1 *vs* Th2), rather than only the quantity (number of tumor-specific CD8 T cells), may lead to future breakthroughs in cancer immunotherapy.

## Methods

### Patients and Lung Tissue Samples

Included NSCLC patients (n=11) underwent surgery by lobectomy, bilobectomy, pneumonectomy or sleeve lobectomy, at the Department of Cardiothoracic Surgery, Oslo University Hospital, Ullevål hospital, Oslo, Norway. The diagnosis of lung cancer was based on histopathologic criteria, and the TNM stage varied from IA to IIIB. Six patients were diagnosed with adenocarcinoma, four patients with SCC, and one patient with ASC (**Table 1**). After the lung lobe containing the tumor had been surgically extracted from the patient, a tumor sample was collected by cutting through the central area of the tumor. Non-cancerous lung tissue was sampled furthest away from the tumor in the resected lobe and designated ‘distal lung’. The patients were either smokers (n=7), former smokers (n=3) or never smoker (n=1). Smokers refer to patients who were actively smoking at the time of the operation and those who quit smoking <1 year before diagnosis. Former smokers refer to patients who had stopped smoking ≥1 year prior to diagnosis. Included patients had not received any treatment for NSCLC (such as chemotherapy, radiotherapy or immunotherapy) or immunosuppressive therapy, prior to surgery and sample collection.

### Multiplex Chromogenic Immunohistochemistry

Tumor and distal lung tissue samples were fixed in 10% neutral buffered formalin for 24 h and embedded in paraffin. Before staining, 3 µm thick serial sections were generated. Multiplex immunostaining was performed using a Ventana Discovery Ultra automated slide stainer (Ventana Medical System, Roche, Cat. No. 750-601). All steps of the staining procedure are described in **Supplementary Table 7**. After deparaffinization of the sections, heat-induced antigen retrieval was performed by using Cell Conditioning 1 buffer (CC1, Ventana Medical System) for 56 min before incubating with the primary mAbs specific for transcription factors and listed in **Supplementary Table 8**. Next, the tissue sections were incubated with secondary antibodies conjugated with peroxidase (**Supplementary Table 9**). Antibodies bound to transcription factors were then visualized with brown dye using the detection DAB kit with brown (Ventana Medical System). To block for potential binding of subsequently added antibodies to the already applied primary and secondary antibodies, the tissue sections were treated with Ribo CC solution (CC2, Ventana Medical System) at 100°C for 24 min. Next, the staining procedure (except for the deparaffinization and antigen retrieval part) was repeated two times sequentially with anti-CD8 and anti-CD3 mAbs on the same sections. Bound anti-CD8 mAb was visualized with Discovery Purple-HRP kit (Ventana Medical System) and bound anti-CD3 mAb with the Discovery Teal-HRP detection kit (Ventana Medical System). Next, a pan-cytokeratin mAb mixture was added on the same section, and Discovery Yellow AP detection kit (Ventana Medical System) was used to visualize the tumor (and normal) epithelium. Finally, the tissue sections were counterstained with hematoxylin. Isotype- and concentration-matched irrelevant antibodies were used as negative controls (**Supplementary Table 8**). All the reagents used for multiplex IHC, including catalog numbers, are listed in **Supplementary Table 10**.

### Immunofluorescence

Formalin-fixed, paraffin-embedded, 3 µm thick tissue sections were deparaffinized and rehydrated, followed by antigen-retrieval by boiling for 20 min in citrate buffer with pH 6 (Dako, Cat. No. S1699). Triple immunostaining was performed by applying a mixture of the primary antibodies listed in **Supplementary Table 11**. The primary antibodies were diluted in phosphate buffered saline (PBS) with bovine albumin (BioRad, Cat. No. 805090). The sections were incubated with the primary antibodies overnight at 4°C. After washing in PBS with 1% Tween 20 (Sigma-Aldrich, Cat. No. P2287) for 5 min, the sections were incubated for 2 h at room temperature with a mixture of secondary antibodies conjugated to fluorophores (**Supplementary Table 12**). Hoechst was added to the final washing buffer to visualize the cell nuclei. Concentration- and isotype-matched irrelevant antibodies were used as negative controls (**Supplementary Table 11**). The reagents used for immunofluorescence staining are listed in **Supplementary Table 13**.

### Image Analyses of Immunostained Tissue Sections

High-power fields (HPF) images of the tissue sections stained by IHC were captured at a size of 0.1302 mm^2^ (0.42 x 0.31 mm) with an Olympus BX51 microscope, model BX51TF (Olympus, Tokyo, Japan) using a Colorview digital camera (Olympus). Images of the tissue sections labeled with immunofluorescence were captured by an Olympus FV1000/BX61 confocal microscope (Olympus). For quantitative analysis of tumor tissue, we examined five representative HPF images, selected to contain both tumor epithelium and tumor stroma. Similarly, five representative HPF were selected to quantify cells in non-cancerous distal lung tissue. If a tissue section had areas with low and high abundance of stained cells, both types of areas were considered as representative and included in the analysis. For TLS, between three and five representative HPF images (one per TLS) were selected from stained sections that contained at least three distinct TLS. Sections containing two or fewer areas with TLS were excluded from the quantification of Th cells in TLS. All representative areas were photographed at a magnification of x400 (HPF). Individual cells were identified by positively stained nuclei and Th cells were manually counted using the ImageJ software, version 1.51 (Fiji). The cell counting was performed independently by two scientists (A. F. and L. B.) and all images were analyzed in a blinded fashion. In case of disagreement, the images were re-examined and the investigators reached a consensus. When calculating the number of cells per mm^2^ in distal lung tissue, empty/white areas were excluded.

### Statistics

For quantitative analysis, cell density data represent the mean number of cells per mm^2^ for three to five representative HPF images analyzed for each stained section. The frequency of each Th subset was calculated as the percentage of the total number of CD3^+^CD8^-^ cells. To determine whether differences between Th subsets in a particular microanatomical region were statistically significant, we used parametric one-way analysis of variance (ANOVA) and Bonferroni´s multiple comparisons *post-hoc* test. To compare the four microanatomical regions for a specific Th subset, we used non-parametric Kruskal-Wallis analysis of variance and post-hoc Dunn´s multiple comparison test. For correlation analysis, Spearman´s Rank Correlation Coefficient was used. All statistical analyses were carried out using GraphPad Prism version 8, except for the correlation analysis that was performed using IBM SPSS version 26. Differences at p<0.05 were considered statistically significant.

## Data Availability Statement

The original contributions presented in the study are included in the article/**Supplementary Material**. Further inquiries can be directed to the corresponding author.

## Ethics Statement

The studies involving human participants were reviewed and approved by The Norwegian Regional Ethical Committee (ref: S-05307). The patients/participants provided their written informed consent to participate in this study.

## Author Contributions

AF performed the experiments, counted the cells, analysed the data, prepared the figures and wrote the manuscript. LB counted the cells. CH analysed and discussed the data. HA, PW, ÅH, and OB provided material from lung cancer patients. IØ analysed and discussed the data and contributed to writing the manuscript. AC designed, supervised and evaluated the experiments, and wrote the manuscript. All authors participated in revising the manuscript and approved the final version.

## Funding

This work was supported by The Research Council of Norway (grant no. 262814), the Norwegian Cancer Society (grant no. 198040), the South-Eastern Norway Regional Health Authority (grant no. 2018046), and by The Research Council of Norway through its Centres of Excellence scheme, project number 262613.

## Conflict of Interest

The authors declare that the research was conducted in the absence of any commercial or financial relationships that could be construed as a potential conflict of interest.

## Publisher’s Note

All claims expressed in this article are solely those of the authors and do not necessarily represent those of their affiliated organizations, or those of the publisher, the editors and the reviewers. Any product that may be evaluated in this article, or claim that may be made by its manufacturer, is not guaranteed or endorsed by the publisher.

## Abbreviations

ASC, adenosquamous carcinoma; BCL6, B-cell lymphoma 6; FFPE, formalin-fixed paraffin-embedded; FOXP3, forkhead box P3; HPF, high-power field; IFN-γ, interferon-γ; IHC, immunohistochemistry; mAb, monoclonal antibody; NSCLC, non-small cell lung cancer; ROR-γt, retinoic-acid-receptor-related orphan receptor -γt; SCC, squamous cell carcinoma**;** T-bet, T-box expressed in T cells; Th, T helper; Tfh, T follicular helper; Treg, regulatory T; TLS, tertiary lymphoid structure.
