# Supplementary material for: The Immune Landscape of Human Primary Lung Tumors Is Th2 Skewed
Source: Front Immunol. 2021 Nov 18;12:764596. doi: 10.3389/fimmu.2021.764596 (PMC8637168; doi:10.3389/fimmu.2021.764596)
Supplement: Supplementary file 1 [file DataSheet_1.pdf]

Supplementary Material

The immune landscape of human primary lung tumors is Th2 skewed

Astri Frafjord *et al.*

Table of Contents

Supplementary Table 1. Correlation analysis of Th and $CD8^+$ T cell densities (cells/mm^2) in tumor stroma and tumor epithelium from 11 NSCLC patients....2
Supplementary Table 2. Correlation analysis of Th subsets (as percent of the total number of $CD3^+CD8^-$ T cells) and $CD8^+$ T cell densities (cells/mm^2) in tumor stroma and tumor epithelium from 11 NSCLC patients....3
Supplementary Table 3. Correlation analysis of densities (cells/mm^2) of Th cell subsets and $CD8^+$ T cells (cells/mm^2) in tertiary lymphoid structures (TLS) from 8 NSCLC patients....4
Supplementary Table 4. Correlation analysis of Th subsets (as percent of the total number of $CD3^+CD8^-$ T cells) and $CD8^+$ T cell densities (cells/mm^2) in tertiary lymphoid structures (TLS) from 8 NSCLC patients....5
Supplementary Table 5. Correlation analysis of densities (cells/mm^2) of Th cell subsets and $CD8^+$ T cells in distal lung from 9 NSCLC patients....6
Supplementary Table 6. Correlation analysis of Th cell subsets (as percent of the total number of $CD3^+CD8^-$ T cells) and $CD8^+$ T cell densities (cells/mm^2) in distal lung from 9 NSCLC patients....7
Supplementary Table 7. Protocols for multiplex chromogenic IHC for detection of Th cell subsets in human tumors....8
Supplementary Table 8. List of primary monoclonal antibodies (mAbs) used to detect T cell subsets and lung carcinoma cells by IHC....9
Supplementary Table 9. List of secondary antibodies used for IHC....9
Supplementary Table 10. List of reagents used for multiplex chromogenic IHC 10
Supplementary Table 11. List of primary antibodies used for immunofluorescence....11
Supplementary Table 12. List of secondary antibodies used for immunofluorescence....11
Supplementary Table 13. List of reagents used for immunofluorescence....11
Supplementary Figure 1. Validation of multiplex IHC of Th1, Th2 and Th17 cell subsets using positive tissue controls....12
Supplementary Figure 2. Validation of multiplex IHC of Tfh and Treg cell subsets using positive tissue controls....13
Supplementary Figure 3. Isotype-matched antibodies with irrelevant specificity as controls for multiplex IHC of Th1 and Th2 subsets 14
Supplementary Figure 4. Isotype-matched antibodies with irrelevant specificity as controls for multiplex IHC of of Treg, Th17 and Tfh subsets 15
Supplementary Figure 5. Validation of the anti-T-bet mAb using another clone 16
Supplementary Figure 6. Validation of the anti-BCL6 mAb using another clone....17
Supplementary Figure 7. Detection of Th1 and Th2 cells by immunofluorescence staining of chemokine receptors in NSCLC tumors 18
Supplementary Figure 8. Detection of Th1 and Th2 cells by immunofluorescence staining of chemokine receptors in tertiary lymphoid structures (TLS)....19
Supplementary Figure 9. Comparison of the number of intratumoral Th1 and Th2 cells detected by chromogenic IHC vs. immunofluorescence....20
Supplementary Figure 10. Comparison of the number of Th1 and Th2 cells detected by chromogenic IHC vs. immunofluorescence in tertiary lymphoid structures (TLS)....21
Supplementary Figure 11. Isotype-matched antibodies with irrelevant specificity as controls for immunofluorescence staining....22

Supplementary Table 1. Correlation analysis of Th and CD8^{+} T cell densities (cells/mm^2) in tumor stroma and tumor epithelium from 11 NSCLC patients[#]

Correlations

			Th1 stroma (cells/mm^2)	Th1 epithelium (cells/mm^2)	Th2 stroma (cells/mm^2)	Th2 epithelium (cells/mm^2)	Th17 stroma (cells/mm^2)	Th17 epithelium (cells/mm^2)	Treg stroma (cells/mm^2)	Treg epithelium (cells/mm^2)	Tfh stroma (cells/mm^2)	Tfh epithelium (cells/mm^2)	CD8 stroma (cells/mm^2)	CD8 epithelium (cells/mm^2)
Spearman´s rho	Th1 stroma (cells/mm^2)	Correlation	1.000	.610*	.600	.473	.165	.000	.373	.210	.624*	.405	.718*	.691*
		P value	.	.046	.051	.142	.627	1.000	.259	.536	.040	.217	.013	.019
		N	11	11	11	11	11	11	11	11	11	11	11	11
	Th1 epithelium (cells/mm^2)	Correlation		1.000	.753**	.839**	.308	.000	.200	.459	.760**	.479	.191	.648*
		P value		.	.007	.001	.357	1.000	.555	.156	.007	.136	.574	.031
		N		11	11	11	11	11	11	11	11	11	11	11
	Th2 stroma (cells/mm^2)	Correlation			1.000	.818**	.312	.300	.473	.579	.780**	.370	.173	.527
		P value			.	.002	.350	.370	.142	.062	.005	.263	.612	.096
		N			11	11	11	11	11	11	11	11	11	11
	Th2 epithelium (cells/mm^2)	Correlation				1.000	.055	-.200	.118	.784**	.670*	.202	.200	.736**
		P value				.	.872	.555	.729	.004	.024	.551	.555	.010
		N				11	11	11	11	11	11	11	11	11
	Th17 stroma (cells/mm^2)	Correlation					1.000	.505	.688*	-.009	.519	.758**	-.064	.055
		P value					.	.113	.019	.979	.102	.007	.851	.872
		N					11	11	11	11	11	11	11	11
	Th17 epithelium (cells/mm^2)	Correlation						1.000	.400	-.301	.303	.509	-.300	-.400
		P value						.	.223	.369	.365	.110	.370	.223
		N						11	11	11	11	11	11	11
	Treg stroma (cells/mm^2)	Correlation							1.000	.050	.404	.457	.236	.045
		P value							.	.884	.218	.158	.484	.894
		N							11	11	11	11	11	11
	Treg epithelium (cells/mm^2)	Correlation								1.000	.474	-.093	.137	.761**
		P value								.	.141	.786	.689	.007
		N								11	11	11	11	11
	Tfh stroma (cells/mm^2)	Correlation									1.000	.548	.083	.624*
		P value									.	.081	.809	.040
		N									11	11	11	11
	Tfh epithelium (cells/mm^2)	Correlation										1.000	.237	.173
		P value										.	.483	.610
		N										11	11	11
	CD8 stroma (cells/mm^2)	Correlation											1.000	.536
		P value											.	.089
		N											11	12
	CD8 epithelium (cells/mm^2)	Correlation												1.000
		P value												.
		N												11

# Spearman´s Rank Correlation Coefficient in IBM SPSS version 26 was used to assess correlations between cell densities of individual Th subsets and CD8^{+} T cells in tumor epithelium and tumor stroma. Value of 1 or -1 represents a perfect correlation, whereas 0 indicates no association.

* p<0.05 level (2-tailed) highlighted in cyan, ** p<0.01 level (2-tailed) highlighted in yellow

Supplementary Table 2. Correlation analysis of Th subsets (as percent of the total number of $CD3^{+}CD8^{-}$ T cells) and $CD8^{+}$ T cell densities (cells/mm^2) in tumor stroma and tumor epithelium from 11 NSCLC patients[#]

Correlations

			Th1 stroma (%)	Th2 stroma (%)	Th17 stroma (%)	Treg stroma (%)	Tfh stroma (%)	CD8 epithelium (cells/mm^2)	CD8 stroma (cells/mm^2)
Spearman`s rho	Th1 stroma (%)	Correlation	1.000	-.536	-.248	-.436	.183	.627*	.845**
		P value	.	.089	.463	.180	.589	.039	.001
		N	11	11	11	11	11	11	11
	Th2 stroma (%)	Correlation		1.000	-.128	.827**	-.110	-.091	-.627*
		P value		.	.707	.002	.747	.790	.039
		N		11	11	11	11	11	11
	Th17 stroma (%)	Correlation			1.000	-.321	.509	-.046	-.239
		P value			.	.336	.110	.893	.480
		N			11	11	11	11	11
	Treg stroma (%)	Correlation				1.000	-.440	-.100	-.418
		P value				.	.175	.770	.201
		N				11	11	11	11
	Tfh stroma (%)	Correlation					1.000	.477	-.092
		P value					.	.138	.788
		N					11	11	11
	CD8 epithelium (cells/mm^2)	Correlation						1.000	.536
		P value						.	.089
		N						11	11
	CD8 stroma (cells/mm^2)	Correlation							1.000
		P value							.
		N							11

[#] Spearman´s Rank Correlation Coefficient in IBM SPSS version 26 was used to assess the correlations between the indicated T cell populations. Value of 1 or -1 represents a perfect correlation, whereas 0 indicates no association.

* $p<0.05$ level (2-tailed) highlighted in cyan

** $p<0.01$ level (2-tailed) highlighted in yellow

Supplementary Table 3. Correlation analysis of densities (cells/mm^2) of Th cell subsets and CD8$^+$ T cells in tertiary lymphoid structures (TLS) from 8 NSCLC patients[#]

Correlations

			Th1 TLS (cells/mm^2)	Th2 TLS (cells/mm^2)	Th17 TLS (cells/mm^2)	Treg TLS (cells/mm^2)	Tfh TLS (cells/mm^2)	CD8 TLS (cells/mm^2)
Spearman´s rho	Th1 TLS (cells/mm^2)	Correlation	1.000	.357	-.214	.071	.167	.690
		P value	.	.385	.610	.867	.693	.058
		N	8	8	8	8	8	8
	Th2 TLS (cells/mm^2)	Correlation		1.000	.262	.381	.595	.238
		P value		.	.531	.352	.120	.570
		N		8	8	8	8	8
	Th17 TLS (cells/mm^2)	Correlation			1.000	.500	.119	.214
		P value			.	.207	.779	.610
		N			8	8	8	8
	Treg TLS (cells/mm^2)	Correlation				1.000	.667	.214
		P value				.	.071	.610
		N				8	8	8
	Tfh TLS (cells/mm^2)	Correlation					1.000	.095
		P value					.	.823
		N					8	8
	CD8 TLS (cells/mm^2)	Correlation						1.000
		P value						.
		N						8

[#] Spearman´s Rank Correlation Coefficient in IBM SPSS version 26 was used to assess the correlation between the indicated T cell populations in tertiary lymphoid structures. Value of 1 or -1 represents a perfect correlation, whereas 0 indicates no association.

None of the analyses reported in the table above had a p value <0.05.

Supplementary Table 4. Correlation analysis of Th subsets (as percent of the total number of $CD3^+CD8^-$ T cells) and $CD8^+$ T cell densities (cells/mm^2) in tertiary lymphoid structures (TLS) from 8 NSCLC patients[#]

Correlations

			Th1 TLS (%)	Th2 TLS (%)	Th17 TLS (%)	Treg TLS (%)	Tfh TLS (%)	CD8 TLS (cells/mm^2)
Spearman´s rho	Th1 TLS (%)	Correlation	1.000	-.095	-.371	.167	-.286	.619
		P value	.	.823	.365	.693	.493	.102
		N	8	8	8	8	8	8
	Th2 TLS (%)	Correlation		1.000	.240	-.810*	.190	.143
		P value		.	.568	.015	.651	.736
		N		8	8	8	8	8
	Th17 TLS (%)	Correlation			1.000	-.036	-.012	.371
		P value			.	.933	.978	.365
		N			8	8	8	8
	Treg TLS (%)	Correlation				1.000	-.071	.167
		P value				.	.867	.693
		N				8	8	8
	Tfh TLS (%)	Correlation					1.000	.143
		P value					.	.736
		N					8	8
	CD8 TLS (cells/mm^2)	Correlation						1.000
		P value						.
		N						8

# Spearman´s Rank Correlation Coefficient in IBM SPSS version 26 was used to assess the correlation between percentage of the individual Th cell subsets and density of $CD8^+$ T cells in tertiary lymphoid structures. Value of 1 or -1 represents a perfect correlation, whereas 0 indicates no association.

* $p<0.05$ level (2-tailed) highlighted in cyan

Supplementary Table 5. Correlation analysis of densities (cells/mm^2) of Th cell subsets and CD8$^+$ T cells in distal lung from 9 NSCLC patients[#]

Correlations

			Th1 distal lung (cells/mm^2)	Th2 distal lung (cells/mm^2)	Th17 distal lung (cells/mm^2)	Treg distal lung (cells/mm^2)	Tfh distal lung (cells/mm^2)	CD8 distal lung (cells/mm^2)
Spearman´s rho	Th1 distal lung (cells/mm^2)	Correlation	1.000	-.322	.276	.483	.797**	.600
		P value	.	.398	.472	.187	.010	.088
		N	9	9	9	9	9	9
	Th2 distal lung (cells/mm^2)	Correlation		1.000	-.562	-.237	-.362	-.661
		P value		.	.115	.539	.338	.053
		N		9	9	9	9	9
	Th17 distal lung (cells/mm^2)	Correlation			1.000	-.075	.136	.293
		P value			.	.847	.727	.444
		N			9	9	9	9
	Treg distal lung (cells/mm^2)	Correlation				1.000	.475	.233
		P value				.	.197	.546
		N				9	9	9
	Tfh distal lung (cells/mm^2)	Correlation					1.000	.475
		P value					.	.197
		N					9	9
	CD8 distal lung (cells/mm^2)	Correlation						1.000
		P value						.
		N						9

[#] Spearman´s Rank Correlation Coefficient in IBM SPSS version 26 was used to assess the correlation between densities (cells/mm^2) of the indicated T cell populations in distal lung tissue. Value of 1 or -1 represents a perfect correlation, whereas 0 indicates no association.

** $p<0.01$ level (2-tailed) highlighted in yellow

Supplementary Table 6. Correlation analysis of Th cell subsets (as percent of the total number of $CD3^+CD8^-$ T cells) and $CD8^+$ T cell densities (cells/mm^2) in distal lung from 9 NSCLC patients[#]

Correlations

			Th1 distal lung (%)	Th2 distal lung (%)	Th17 distal lung (%)	Treg distal lung (%)	Tfh distal lung (%)	CD8 distal lung (cells/mm^2)
Spearman´s rho	Th1 distal lung (%)	Correlation	1.000	.238	.720*	.533	-.373	-.317
		P value	.	.537	.029	.139	.323	.406
		N	9	9	9	9	9	9
	Th2 distal lung (%)	Correlation		1.000	-.209	.179	-.355	-.698*
		P value		.	.589	.645	.349	.037
		N		9	9	9	9	9
	Th17 distal lung (%)	Correlation			1.000	.268	-.213	-.059
		P value			.	.486	.583	.881
		N			9	9	9	9
	Treg distal lung (%)	Correlation				1.000	.390	-.550
		P value				.	.300	.125
		N				9	9	9
	Tfh distal lung (%)	Correlation					1.000	.051
		P value					.	.897
		N					9	9
	CD8 distal lung (cells/mm^2)	Correlation						1.000
		P value						.
		N						9

[#] Spearman´s Rank Correlation Coefficient in IBM SPSS version 26 was used to assess the correlation in distal lung between the percentages of individual Th cell subsets and the density of $CD8^+$ T cells. Value of 1 or -1 represents a perfect correlation, whereas 0 indicates no association.

* $p<0.05$ level (2-tailed) highlighted in cyan

Supplementary Table 7. Protocols for multiplex chromogenic IHC for detection of Th cell subsets in human tumors

STEPS	Th1 cell stain	Th2 cell stain (clone L50-816)	Th2 cell stain (clone D13C9)	Treg cell stain	Th17 cell stain	Tfh cell stain
1. Deparaffinization cycle (time)	manual	3 (24 min)	3 (24 min)	3 (24 min)	3 (24 min)	3 (24 min)
2. Pretreatment/ antigen retrieval (time)	Tris-EDTA pH=9 (20 min) manual	CC1 (56 min)	CC1 (56 min)	CC1 (56 min)	CC1 (56 min)	CC1 (56 min)
3. Peroxidase block (time)	Peroxidase-blocking (5 min) manual	DISCOVERY Inhibitor (8 min)	DISCOVERY Inhibitor (8 min)	DISCOVERY Inhibitor (8 min)	DISCOVERY Inhibitor (8 min)	DISCOVERY Inhibitor (8 min)
4. Peroxidase block (time)	-	Inhibitor CM (8 min)	Inhibitor CM (8 min)	Inhibitor CM (8 min)	Inhibitor CM (8 min)	Inhibitor CM (8 min)
5. Protein block (time)	Protein block (20 min) manual	-	-	-	-	-
6. Primary antibody (time)	T-bet: overnight at 4 °C manual	GATA-3, clone L50-816 (32 min)	GATA-3, clone D13C9 (60 min)	FOXP3 (32 min)	ROR-γt (60 min)	BCL-6 (32 min)
7. Secondary antibody (time)	EnVision™ FLEX/ HRP (30 min) manual	HQ anti-mouse (16 min)	HQ anti-rabbit (20 min)	HQ anti-mouse (16 min)	HQ anti-mouse (20 min)	HQ anti-mouse (16 min)
8. Secondary antibody (time)	-	anti-HQ (16 min)	anti-HQ (20 min)	anti-HQ (16 min)	anti-HQ (16 min)	anti-HQ (16 min)
9. Substrate	HRP	HRP	HRP	HRP	HRP	HRP
10. Chromogen (time)	DAB (5 min) manual	DAB (default)	DAB (default)	DAB (default)	DAB (default)	DAB (default)
11. Ribo CC (CC2) (temperature, time)	-	100 °C, 24 min	100 °C, 24 min	100 °C, 24 min	100 °C, 24 min	100 °C, 24 min
12. Peroxidase block (time)	DISCOVERY Inhibitor (8 min)	-	-	-	-	-
13. Primary antibody (time)	CD8 (32 min)	CD8 (32 min)	CD8 (32 min)	CD8 (32 min)	CD8 (32 min)	CD8 (32 min)
14. Secondary antibody (time)	anti-rabbit OmniMap (16 min)	anti-rabbit OmniMap (16 min)	anti-rabbit OmniMap (16 min)	anti-rabbit OmniMap (16 min)	anti-rabbit OmniMap (16 min)	anti-rabbit OmniMap (16 min)
15. Substrate	HRP	HRP	HRP	HRP	HRP	HRP
16. Chromogen (time)	Purple (32 min)	Purple (32 min)	Purple (32 min)	Purple (32 min)	Purple (32 min)	Purple (32 min)
17. Ribo CC (CC2) (temperature, time)	100 °C, 24 min	100 °C, 24 min	100 °C, 24 min	100 °C, 24 min	100 °C, 24 min	100 °C, 24 min
18. Primary antibody (time)	CD3 (44 min)	CD3 (44 min)	CD3 (44 min)	CD3 (44 min)	CD3 (44 min)	CD3 (44 min)
19. Secondary antibody (time)	anti-rabbit HQ (16 min)	anti-rabbit HQ (16 min)	anti-rabbit HQ (16 min)	anti-rabbit HQ (16 min)	anti-rabbit HQ (16 min)	anti-rabbit HQ (16 min)
20. Secondary antibody (time)	anti-HQ (16 min)	anti-HQ (16 min)	anti-HQ (16 min)	anti-HQ (16 min)	anti-HQ (16 min)	anti-HQ (16 min)
21. Substrate	HRP	HRP	HRP	HRP	HRP	HRP
22. Chromogen (time)	Teal (H_2O_2:32 min) (Act:16 min)	Teal (H_2O_2:32 min) (Act:16 min)	Teal (H_2O_2:32 min) (Act:16 min)	Teal (H_2O_2:32 min) (Act:16 min)	Teal (H_2O_2:32 min) (Act:16 min)	Teal (H_2O_2:32 min) (Act:16 min)
23. Ribo CC (CC2) (temperature, time)	100 °C, 24 min	100 °C, 24 min	100 °C, 24 min	100 °C, 24 min	100 °C, 24 min	100 °C, 24 min
24. Pretreatment/antigen retrieval (time)	Protease 3 (4 min)	Protease 3 (4 min)	Protease 3 (4 min)	Protease 3 (4 min)	Protease 3 (4 min)	Protease 3 (4 min)
25. Primary antibody (time)	Cytokeratin (20 min)	Cytokeratin (20 min)	Cytokeratin (20 min)	Cytokeratin (20 min)	Cytokeratin (20 min)	Cytokeratin (20 min)
26. Secondary antibody (time)	anti-mouse UltraMap (16 min)	anti-mouse UltraMap (16 min)	anti-mouse UltraMap (16 min)	anti-mouse UltraMap (16 min)	anti-mouse UltraMap (16 min)	anti-mouse UltraMap (16 min)
27. Enzyme	AP	AP	AP	AP	AP	AP
28. Chromogen (time)	Yellow (60 min)	Yellow (60 min)	Yellow (60 min)	Yellow (60 min)	Yellow (60 min)	Yellow (60 min)
29. Manual counterstain (hematoxylin)	1 min	1 min	1 min	1 min	1 min	1 min

An autostainer Ventana Discovery Ultra system was used for multiplex immunohistochemistry.

Abbreviations: Act, activator; AP, alkaline phosphatase; CC1, cell conditioning 1; CC2, cell conditioning 2; DAB, 3,3'-diaminobenzidine; HRP, horseradish peroxidase; HQ, hydroxyquinazoline

Supplementary Table 8. List of primary monoclonal antibodies (mAbs) used to detect T cell subsets and lung carcinoma cells by IHC

Specificity of primary antibody	mAb clone	Species/ Isotype	Dilution (μg/ml)	Supplier	Cat. No.	Incubation time	Isotype-matched control mAbs	
							Supplier	Cat. No.
T-bet	4B10	Mouse IgG1	1:700 (0.28)	Santa Cruz Biotechnology	sc-21749	Overnight at 4 °C	Dako	X0931
GATA-3	L50-816	Mouse IgG1	ready to use (1.84)	Roche	760-4897	32 min	Dako	X0931
GATA-3	D13C9	Rabbit IgG	1:100 (5.0)	CellSignalling	5852	60 min	Vector Laboratories	I-1000
ROR-gt	6F3.1	Mouse IgG2a	1:100 (10.0)	EMD Millipore	MABF81	56 min	Dako	X0943
Bcl6	Gi191/A8	Mouse IgG1	ready to use (0.3)	Roche	760-4241	32 min	Dako	X0931
Foxp3	236A/E7	Mouse IgG1	1:100 (5.0)	Invitrogen	14-4777-82	56 min	Dako	X0931
CD3	2GV6	Rabbit monocloncal	ready to use (0.40)	Roche	790-4341	44 min	Vector Laboratories	I-1000
CD8	SP57	Rabbit monoclonal	ready to use (0.35)	Roche	790-4460	32 min	Vector Laboratories	I-1000
Cyto-keratin	AE1/AE3/ PCK26	Mouse IgG1	ready to use (48.0)	Roche	760-2135	20 min	Dako	X0931

Supplementary Table 9. List of secondary antibodies used for IHC

Target of primary antibody	Secondary antibodies	Dilution	Supplier	Cat. No.	Incubation time
T-bet mouse IgG1	EnVision™ FLEX/ HRP	Ready to use	Dako	DM822	30 min
GATA-3 mouse IgG1 (clone L50-816)	1. HQ anti-mouse 2. HRP anti-HQ	Ready to use	Roche	1. 760-4814 2. 760-4820	1. 16 min 2. 16 min
GATA-3 rabbit IgG (clone D13C9)	1. HQ anti-rabbit 2. HRP anti-HQ	Ready to use	Roche	1. 760-4815 2. 760-4820	1. 20 min 2. 20 min
ROR-γt mouse IgG2a	1. HQ anti-mouse 2. HRP anti-HQ	Ready to use	Roche	1. 760-4814 2. 760-4820	1. 20 min 2. 16 min
Bcl6 mouse IgG1	1. HQ anti-mouse 2. HRP anti-HQ	Ready to use	Roche	1. 760-4814 2. 760-4820	1. 16 min 2. 16 min
Foxp3 mouse IgG1	1. HQ anti-mouse 2. HRP anti-HQ	Ready to use	Roche	1. 760-4814 2. 760-4820	1. 16 min 2. 16 min
CD3 rabbit IgG	1. HQ anti-rabbit 2. HRP anti-HQ	Ready to use	Roche	1. 760-4815 2. 760-4820	1. 16 min 2. 16 min
CD8 rabbit IgG	DISCOVERY anti-rabbit OmniMap HRP	Ready to use	Roche	760-4311	16 min
Cytokeratin mouse IgG1	DISCOVERY anti-mouse UltraMap AP	Ready to use	Roche	760-4312	16 min

Abbreviations: AP, alkaline phosphatase; HRP, horseradish peroxidase; NP, hapten nitropyrazole; HQ, hydroxyquinazoline; mAb, monoclonal antibody

Supplementary Table 10. List of reagents used for multiplex chromogenic IHC

Regents used in IHC	Supplier	Cat. No.
Tween 20	Sigma-Aldrich	P2287
HyClone Dulbecco's Phosphate Buffered Saline: Powder	GE Healthcare Life Sciences	SH30013.03
CC1 (cell conditioning 1)	Roche	950-124
Ribo CC (CC2)	Roche	760-107
Tris-EDTA (pH=9)	Dako, Agilent	K8004
Protease 3	Roche	760-2020
DISCOVERY Inhibitor	Roche	760-4840
Inhibitor CM	Roche	760-124
EnVision™ FLEX Peroxidase-blocking Reagent	Dako, Agilent	DM821
Protein block	Dako, Agilent	X0909
DISCOVERY DAB Map Detection kit	Roche	760-124
DISCOVERY Purple Detection kit	Roche	760-229
DISCOVERY Teal Detection kit	Roche	760-247
DISCOVERY Yellow Detection kit	Roche	760-239
DAKO Liquid DAB+ Substrate Chromogen System	Dako, Agilent	K3468

Supplementary Table 11. List of primary antibodies used for immunofluorescence

Primary antibodies	Clone	Species/isotype	Dilution (μg/ml)	Supplier	Cat. No.	Antigen retrieval	Isotype-matched control mAbs	
							Supplier	Cat. No.
**CCR4**	Polyclonal	Rabbit IgG	1:50 (2.0)	Sigma-Aldrich	HPA031613	Citrate (pH 6.0)	Vector Laboratories	I-1000
**CXCR3**	49801	Mouse IgG1	1:200 (2.5)	R&D systems	MAB160-100	Citrate (pH 6.0)	Dako	X0931
**CD3**	2GV6	Mouse IgG1	ready to use (0.40)	Roche	790-4341	Citrate (pH 6.0)	Dako	X0931
**CD3**	F7.2.38	Rabbit IgG	1:50 (5.0)	Abcam	ab17143	Citrate (pH 6.0)	Vector Laboratories	I-1000
**CD8**	4B11	Mouse IgG2b	1:50 (5.0)	Thermo Scientific	MA1-80231	Citrate (pH 6.0)	Biolegend	400301

Supplementary Table 12. List of secondary antibodies used for immunofluorescence

Target of primary antibody	Species/ isotype for primary antibodies	Fluorochrome	Species	Dilution	Supplier	Cat. No.
**CCR4**	Rabbit IgG	Alexa Fluor 647	Donkey	1:500	Molecular Probes	A-31573
**CXCR3**	Mouse IgG1	Alexa Fluor 488	Goat	1:500	Molecular Probes	A-21121
**CD3**	Mouse IgG1	Alexa Fluor 488	Goat	1:500	Molecular Probes	A-21121
**CD3**	Rabbit IgG	Alexa Fluor 647	Donkey	1:500	Molecular Probes	A-31573
**CD8**	Mouse IgG2b	Alexa Fluor 555	Goat	1:500	Molecular Probes	A-21147

Supplementary Table 13. List of reagents used for immunofluorescence

Regents used in IHC	Supplier	Cat. No.
Citrate buffer pH 6	Dako, Agilent	S1699
Protein block	Dako, Agilent	X0909
Bovine Albumin	BioRad	805090
Tween 20	Sigma-Aldrich	P2287
HyClone Dulbecco's Phosphate Buffered Saline: Powder	GE Healthcare Life Sciences	SH30013.03

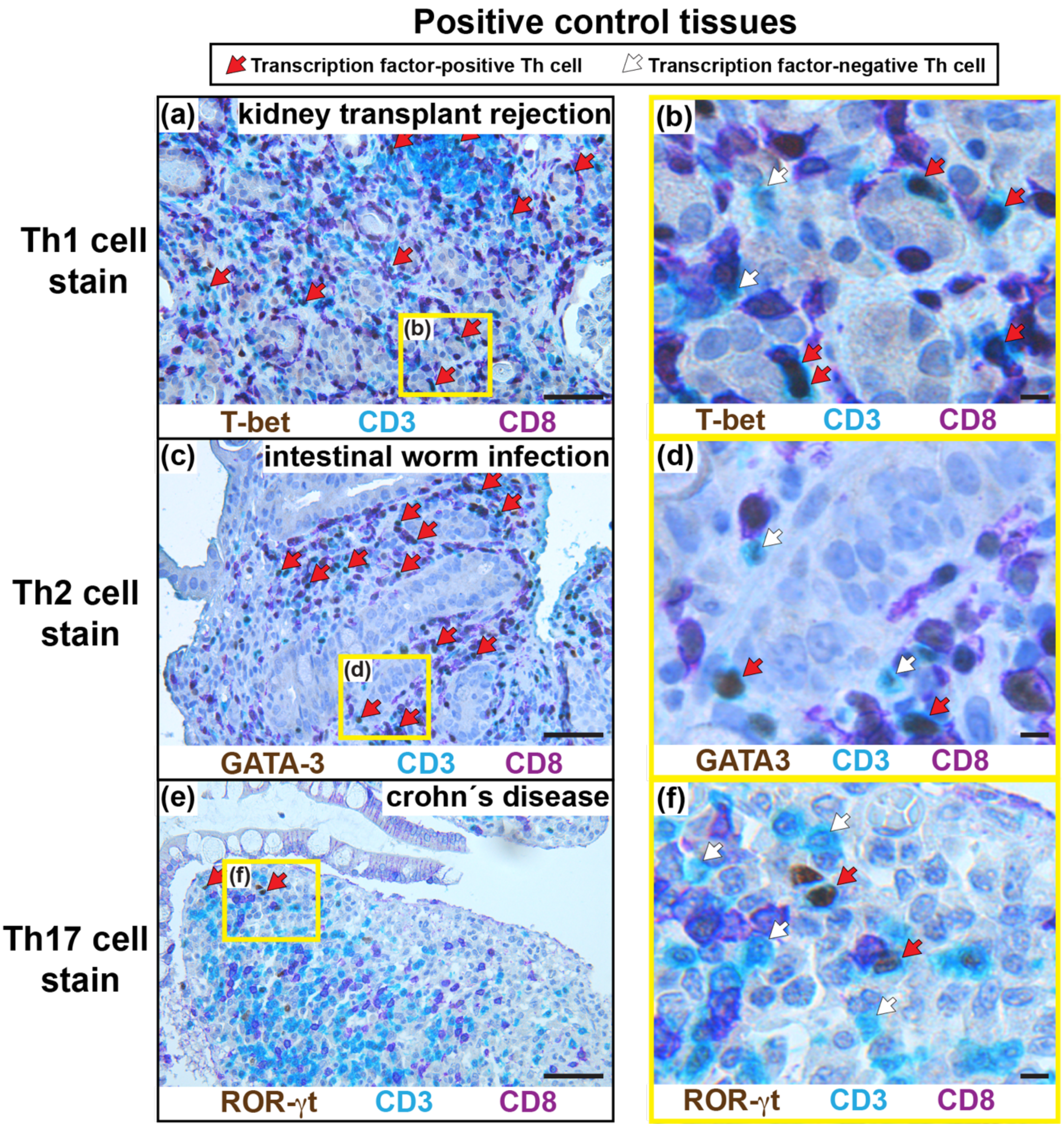

Supplementary Figure 1. Validation of multiplex IHC of Th1, Th2 and Th17 cell subsets using positive tissue controls. Tissues that were expected to contain high numbers of particular T cell subsets were selected and representative images are shown: **(a, b)** tissue of kidney transplant rejection for Th1 cells (T-bet$^+$CD3$^+$CD8$^-$); **(c, d)** intestinal tissue with worm infection for Th2 cells (GATA-3$^+$CD3$^+$CD8$^-$) identified by the anti-GATA-3 mAb clone L50-816; **(e, f)** gut tissue from a patient with Crohn´s disease for Th17 cells (ROR-γt$^+$CD3$^+$CD8$^-$). **(a-f)** T cells were immunostained with anti-CD3 (teal) and anti-CD8 (purple) mAbs, and all Th cells were identified as CD3$^+$CD8$^-$. Th subsets were detected using mAbs against subset-defining transcription factors (brown). Arrows indicate examples of positive and negative cells: red arrows point at transcription factor-positive Th cells (teal cells with brown nuclei), and white arrows point at transcription factor-negative Th cells (teal cells with hematoxylin-stained purplish blue nuclei). The squared areas indicated in yellow in **(a, c, e)** are shown in **(b, d, f)** with higher magnification. Original magnification, 400x. **(a, c, e)** Scale bars, 50 µm. **(b, d, f)** Scale bars, 5 µm.

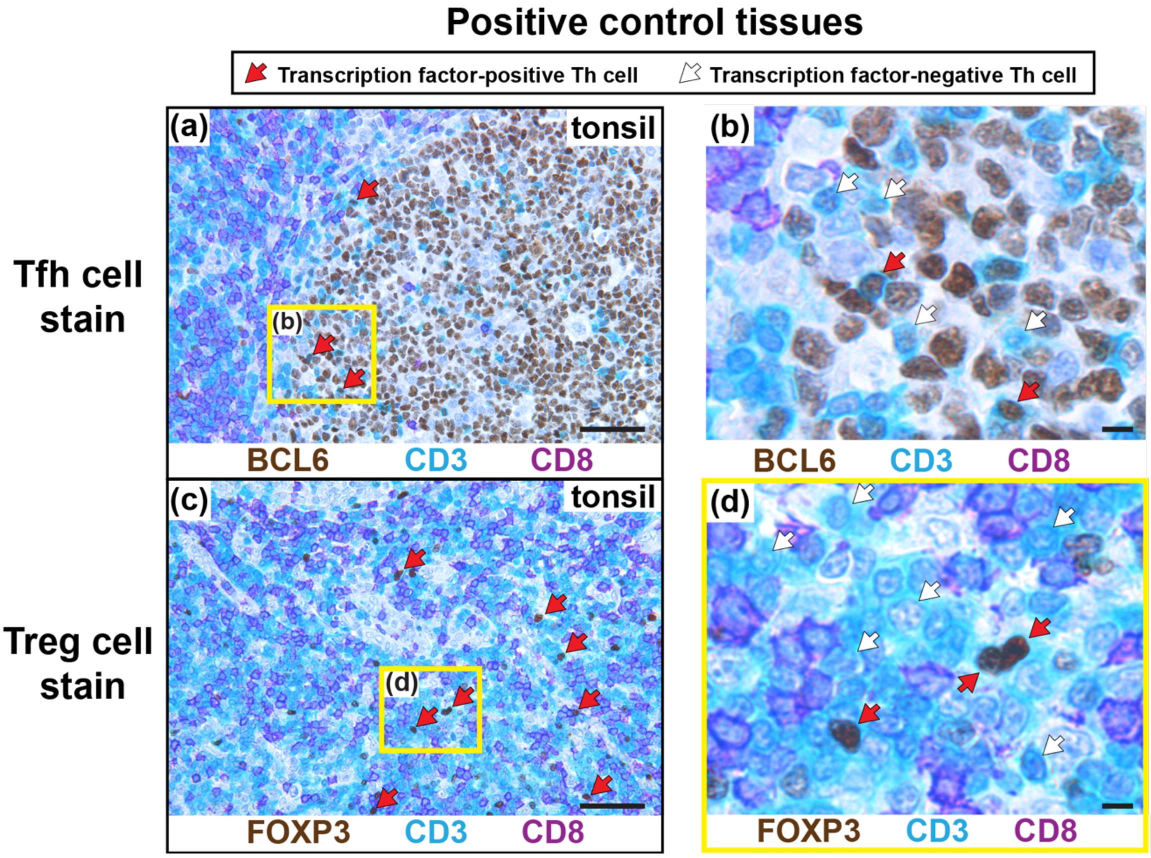

Supplementary Figure 2. Validation of multiplex IHC of Tfh and Treg cell subsets using positive tissue controls. Tonsil tissue that is expected to contain high numbers of Tfh and Treg cells was selected, and representative images are shown. T cells were immunostained with anti-CD3 (teal) and anti-CD8 (purple) mAbs, and all Th cells were identified as $CD3^{+}CD8^{-}$. Th subsets were detected using mAbs against subset-defining transcription factors (brown). Arrows indicate examples of positive and negative cells: red arrows point at transcription factor-positive Th cells (teal cells with brown nuclei), and white arrows point at transcription factor-negative Th cells (teal cells with hematoxylin-stained purplish blue nuclei). **(a, b)** Tfh cells ($BCL6^{+}CD3^{+}CD8^{-}$) in tonsil. **(c, d)** Treg cells ($FOXP3^{+}CD3^{+}CD8^{-}$) in tonsil. The squared areas indicated in yellow in **(a, c)** are shown in **(b, d)** with higher magnification. Original magnification, 400x. **(a, c)** Scale bars, 50 μm. **(b, d)** Scale bars, 5 μm.

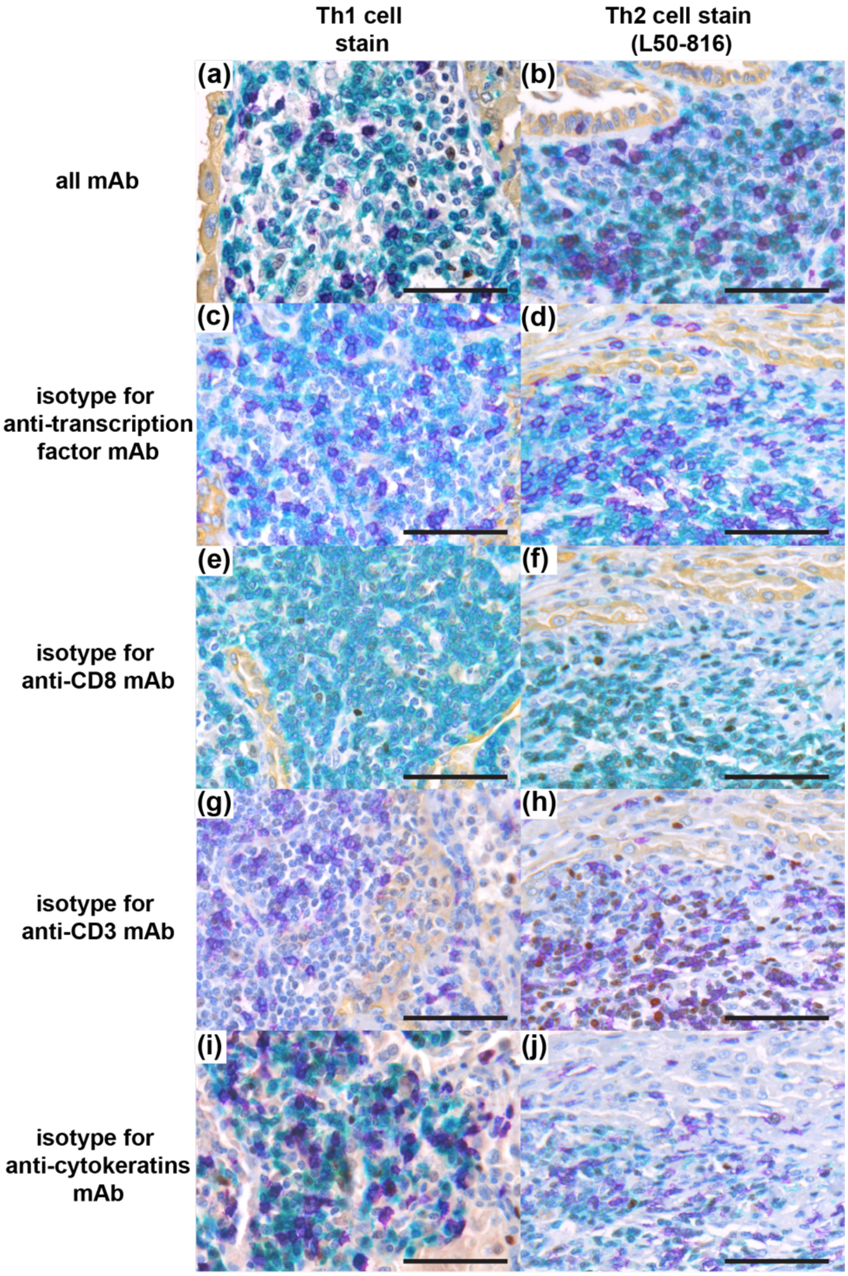

Supplementary Figure 3. Isotype-matched antibodies with irrelevant specificity as controls for multiplex IHC of Th1 and Th2 subsets. (a, b) Positive controls: detection of Th1 cells **(a)** and Th2 cells **(b)** in NSCLC tumors using the established multiplex IHC assay (all antibodies). **(c-j)** Each antibody of the assay was replaced by an isotype-matched control mAb with irrelevant specificity: **(c)** mouse IgG1 mAb for anti-T-bet mAb clone 4B10; **(d)** mouse IgG1 mAb for anti-GATA-3 mAb clone L50-816; **(e, f)** rabbit IgG mAb for anti-CD8 mAb clone SP57; **(g, h)** rabbit IgG mAb for anti-CD3 mAb clone 2GV6; **(i, j)** mouse IgG1 mAb for anti-cytokeratins mAb clones AE1/AE3/PCK26. The representative images shown above illustrate the absence of non-specific background staining of the tumor sections by the isotype-matched control antibodies. Original magnification, 400x. Scale bars, 50 μm.

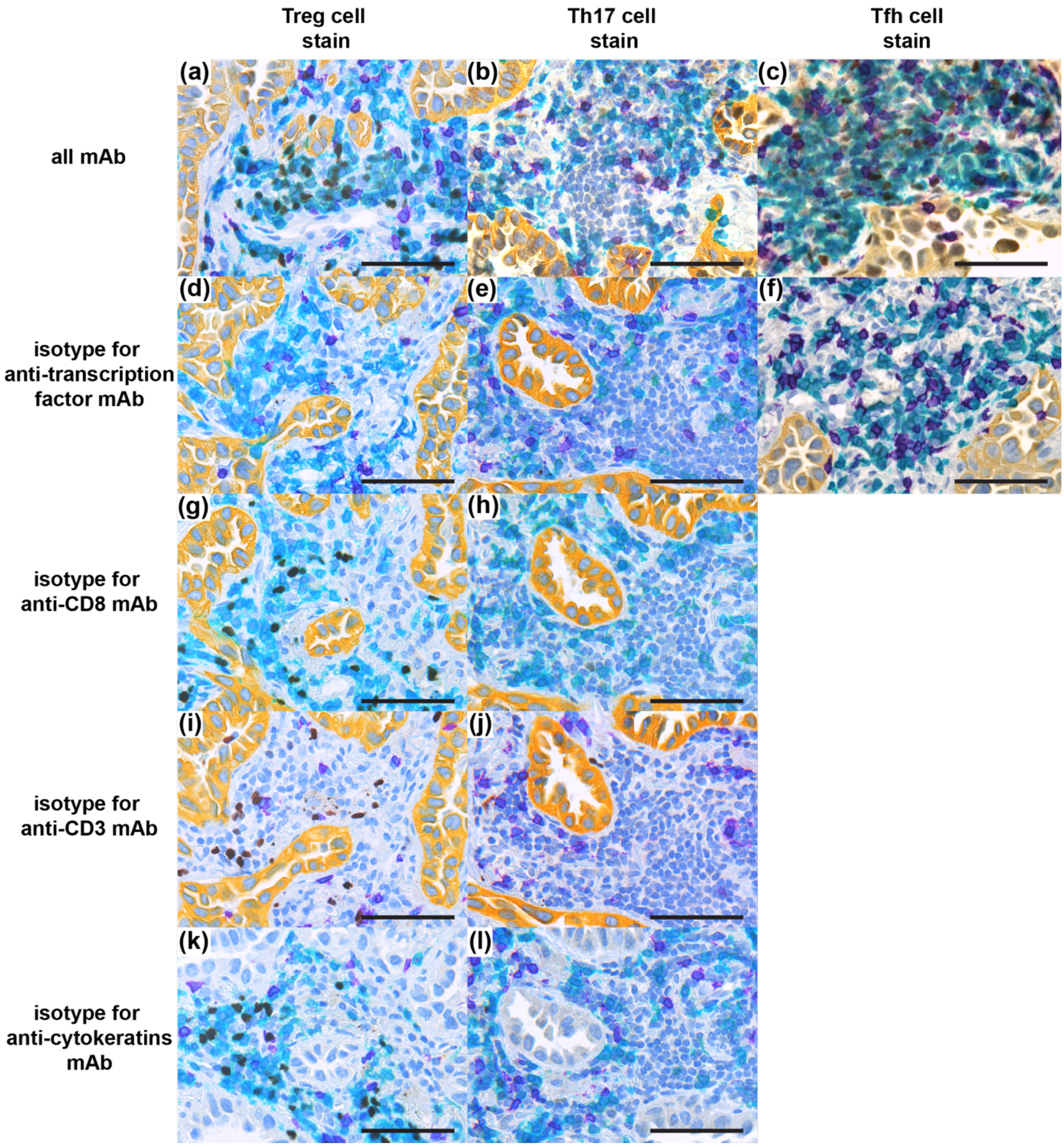

Supplementary Figure 4. Isotype-matched antibodies with irrelevant specificity as controls for multiplex IHC of Treg, Th17 and Tfh subsets. (a, b, c) Positive controls: detection of Treg cells **(a)**, Th17 cells **(b)**, and Tfh cells **(c)** in NSCLC tumors using the established multiplex IHC assay (all antibodies). **(d-l)** Each antibody of the assay was replaced by an isotype-matched control mAb with irrelevant specificity: **(d)** mouse IgG1 mAb for anti-FOXP3 mAb clone 236A/E7; **(e)** mouse IgG2a mAb for anti-ROR-γt mAb clone 6F3.1; **(f)** mouse IgG1 mAb for anti-BCL6 mAb clone Gi191/A8; **(g, h)** rabbit IgG mAb for anti-CD8 mAb clone SP57; **(i, j)** rabbit IgG mAb for anti-CD3 mAb clone 2GV6; **(k, l)** mouse IgG1 mAb for anti-cytokeratins mAb clones AE1/AE3/PCK26. The representative images shown above illustrate the absence of non-specific background staining of the tumor sections by the isotype-matched control antibodies. Original magnification, 400x. Scale bars, 50 µm.

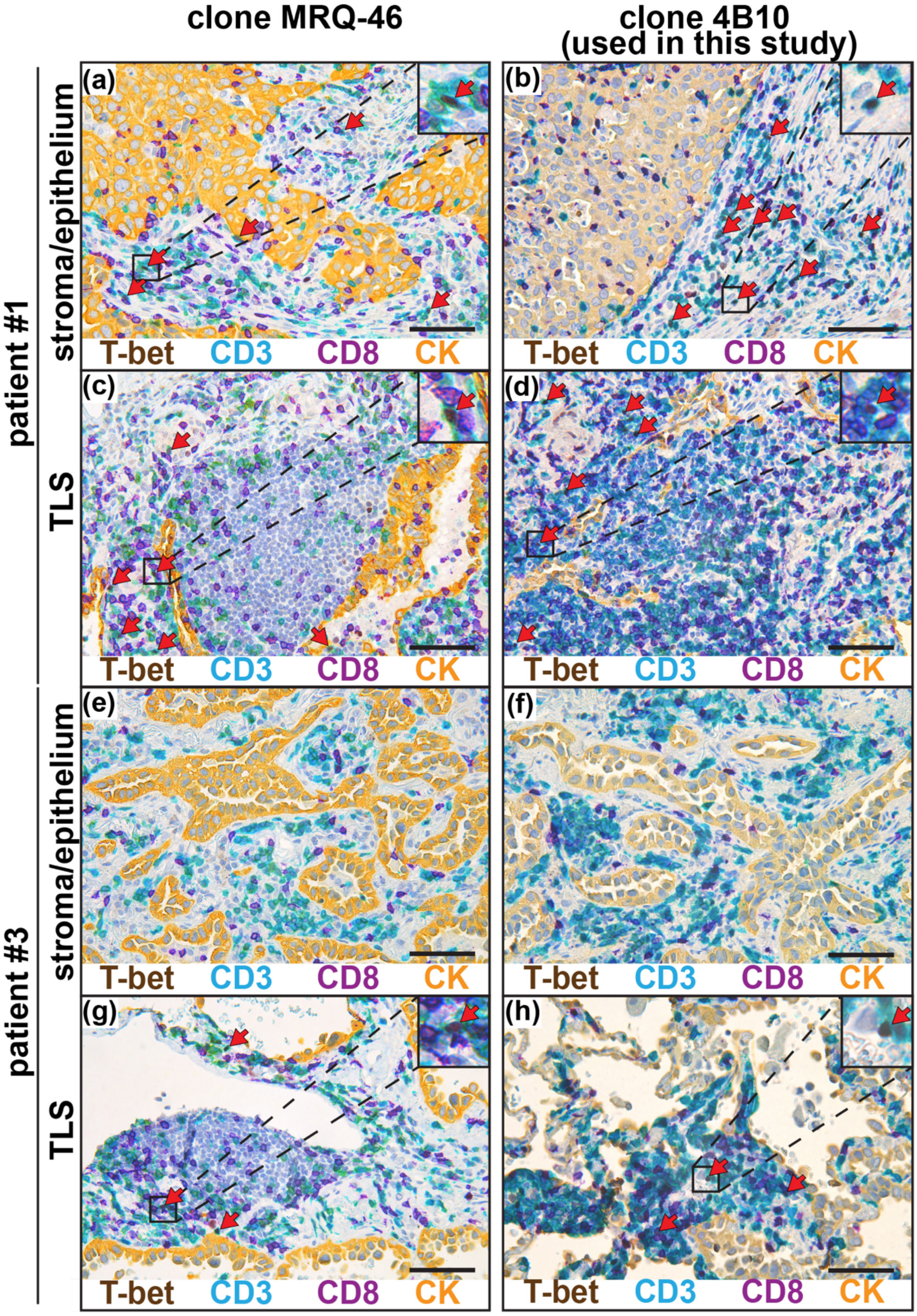

Supplementary Figure 5. Validation of the anti-T-bet mAb using another clone. Representative images of serial immunostained sections from two lung adenocarcinoma patients. Boxed areas are enlarged and presented as corner insets to show positively stained Th cells. Th1 cells identified as teal colored cells with brown nuclei ($T\text{-bet}^{+}CD3^{+}CD8^{-}$) are indicated with red arrows. The tissue sections were incubated with **(a, c, e, g)** the anti-T-bet mAb clone MRQ-46 or **(b, d, f, h)** the anti-T-bet mAb clone 4B10. Clone 4B10 was used for the established multiplex IHC assay. The images shown above reveal that the two anti-T-bet mAb clones detect similar numbers of Th1 cells per mm^2. TLS, tertiary lymphoid structure. CK, cytokeratin. Original magnification, 400x. Scale bars, 50 µm.

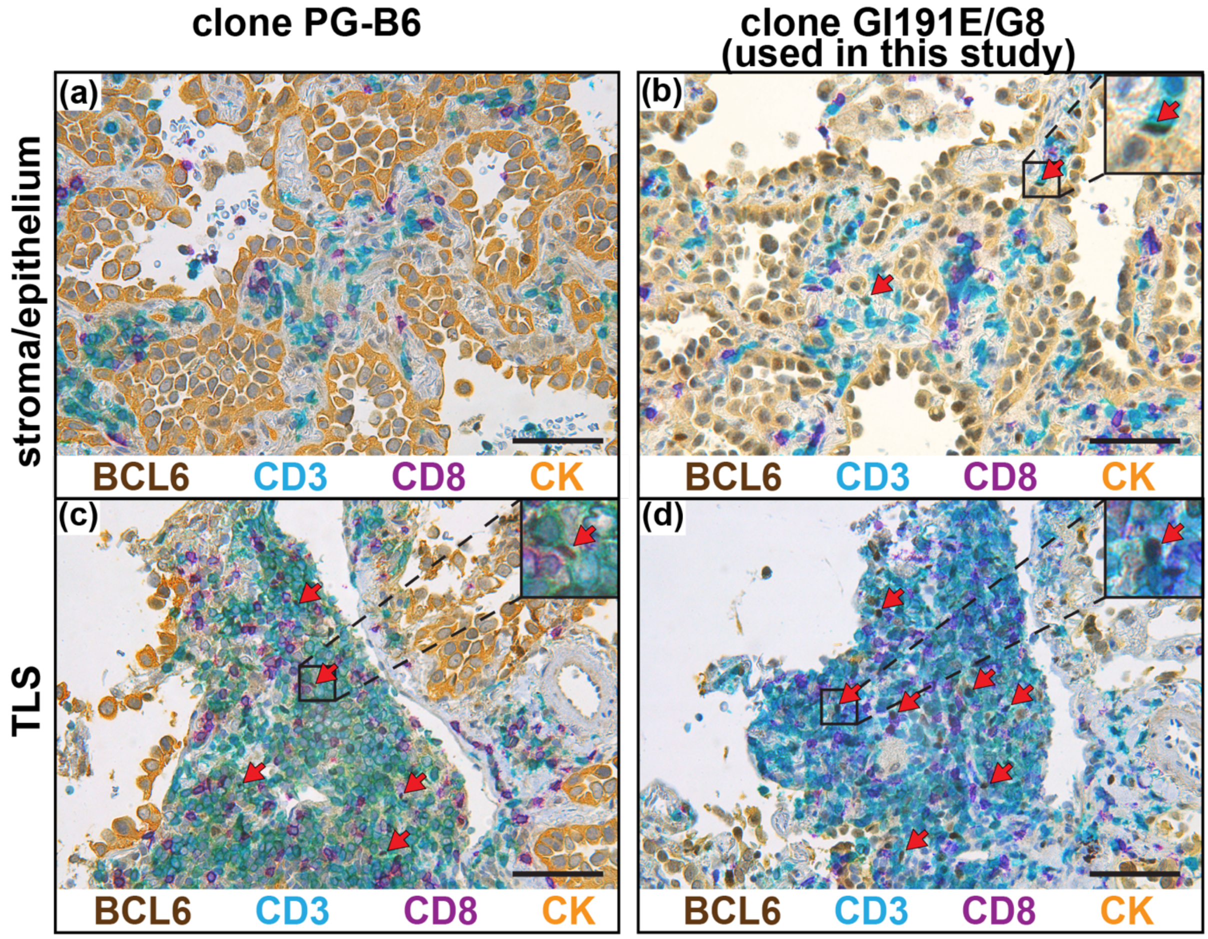

Supplementary Figure 6. Validation of the anti-BCL6 mAb using another clone. (a-d) Representative images of serial immunostained sections from one lung adenocarcinoma patient. Boxed areas are enlarged and presented as corner insets to show positively stained Tfh cells. Tfh cells identified as teal colored cells with brown nuclei ($BCL6^{+}CD3^{+}CD8^{-}$) are indicated with red arrows The tissue sections were incubated with **(a, c)** anti-BCL6 mAb clone PG-B6, or **(b, d)** anti-BCL6 mAb clone GI191E/G8. Clone GI191E/G8 was used for the established multiplex IHC assay. The images shown above reveal that the two anti-BCL6 mAb clones detect similar numbers of Tfh cells per mm^2. TLS, tertiary lymphoid structure. CK, cytokeratin. Original magnification, 400x. Scale bars, 50 µm.

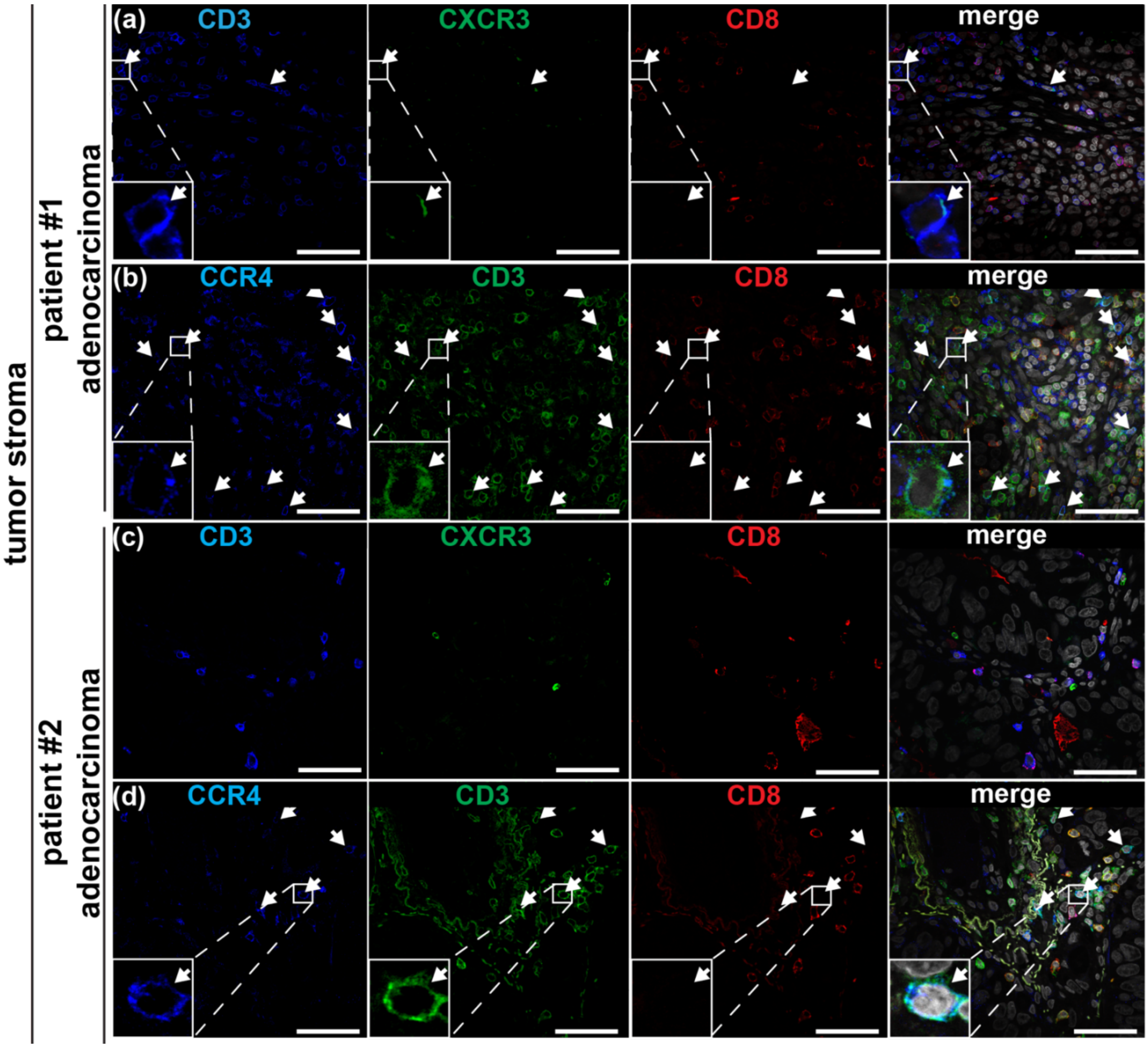

Supplementary Figure 7. Detection of Th1 and Th2 cells by immunofluorescence staining of chemokine receptors in NSCLC tumors. Representative images of immunostained serial sections of tumor tissue from two patients with lung adenocarcinoma. Expression of CXCR3 and CCR4 were used as markers for Th1 and Th2 cells, respectively. **(a, c)** Detection of Th1 cells. Tumor sections were immunostained with anti-CD3 mAb (blue), anti-CXCR3 mAb (green), and anti-CD8 mAb (red). Only low numbers of $CD3^{+}CD8^{-}CXCR3^{+}$ Th1 cells (white arrows) were observed. **(b, d)** Detection of Th2 cells. Tumor sections were immunostained with anti-CCR4 polyclonal rabbit IgG (blue), anti-CD3 mAb (green), and anti-CD8 mAb (red). The observed $CD3^{+}CD8^{-}CCR4^{+}$ Th2 cells are indicated by white arrows. Similar results were observed in tumor tissue from all investigated NSCLC patients (n=3) and are consistent with a predominance of Th2 cells in lung tumors. Original magnification, 600x. Scale bars, 50 µm.

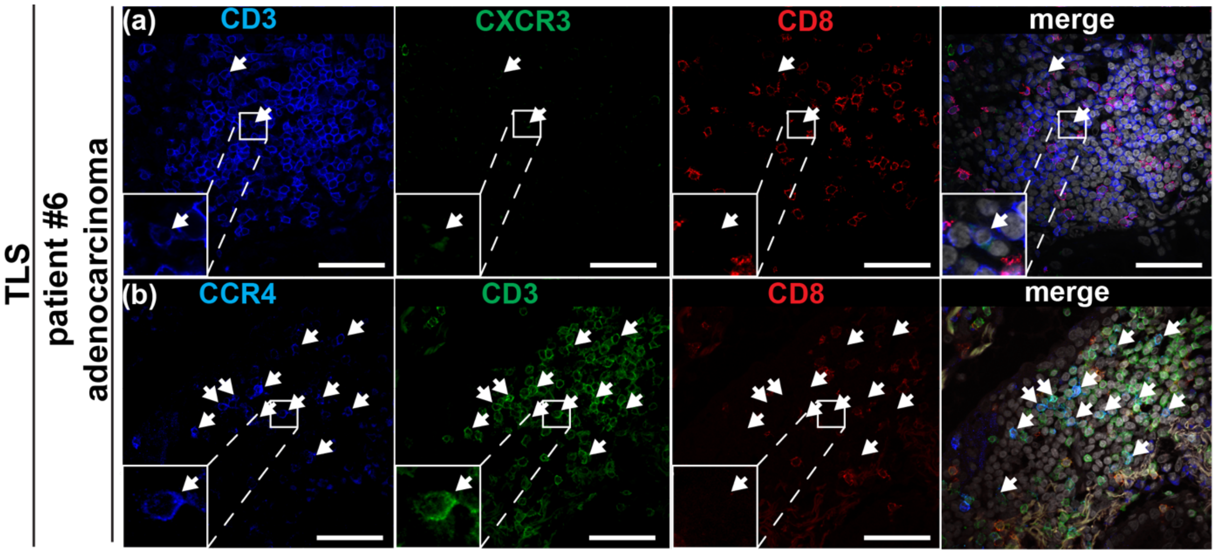

Supplementary Figure 8. Detection of Th1 and Th2 cells by immunofluorescence staining of chemokine receptors in tertiary lymphoid structures (TLS). Representative images of immunostained serial sections of a TLS from one patient with lung adenocarcinoma. Expression of CXCR3 and CCR4 were used as markers for Th1 and Th2 cells, respectively. **(a)** Detection of Th1 cells. The TLS was immunostained with anti-CD3 mAb (blue), anti-CXCR3 mAb (green), and anti-CD8 mAb (red). Only low numbers of $CD3^{+}CD8^{-}CXCR3^{+}$ Th1 cells (white arrows) were observed. **(b)** Detection of Th2 cells. The TLS was immunostained with anti-CCR4 polyclonal rabbit IgG (blue), anti-CD3 mAb (green), and anti-CD8 mAb (red). The observed $CD3^{+}CD8^{-}CCR4^{+}$ Th2 cells are indicated by white arrows. Similar results were observed in TLS from all investigated NSCLC patients (n=3) and are consistent with a predominance of Th2 cells in TLS. Original magnification, 600x. Scale bars, 50 µm.

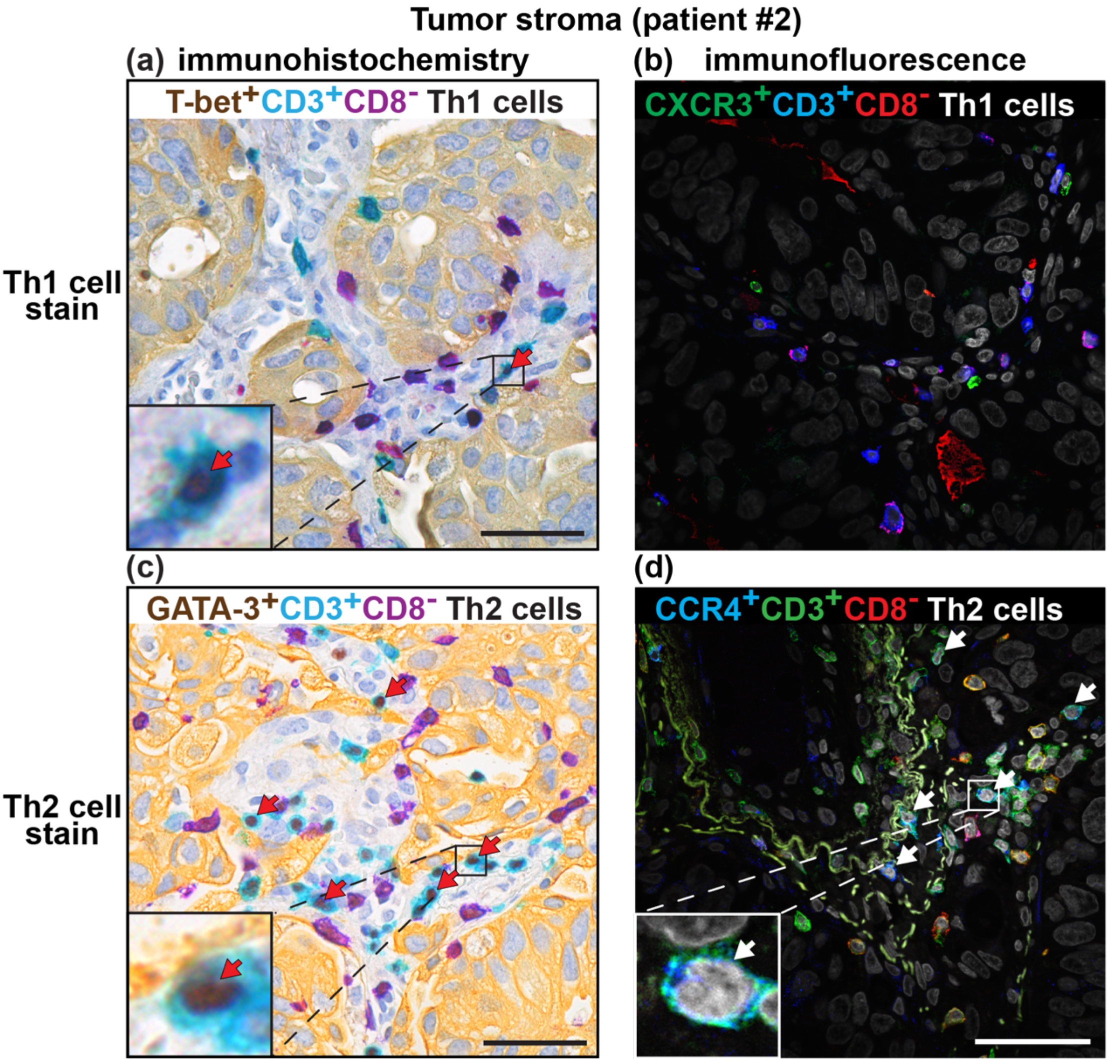

Supplementary Figure 9. Comparison of the number of intratumoral Th1 and Th2 cells detected by chromogenic IHC vs. immunofluorescence. Representative images of immunostained tumor sections from one patient with lung adenocarcinoma (patient #2). Boxed areas are enlarged and presented as corner insets. The positive cells for each Th subset in tumor stroma are indicated by red or white arrows. **(a)** Th1 cells (T-bet^{+}CD3^{+}CD8^{-}) detected by chromogenic IHC. **(b)** Th1 cells (CXCR3^{+}CD3^{+}CD8^{-}) detected by immunofluorescence. **(c)** Th2 cells (GATA-3^{+}CD3^{+}CD8^{-}) detected by chromogenic IHC, using the anti-GATA-3 mAb clone L50-816. **(d)** Th2 cells (CCR4^{+}CD3^{+}CD8^{-}) detected by immunofluorescence. Similar results were obtained from all three investigated NSCLC patients. Original magnification, 600x. Scale bars, 50 µm.

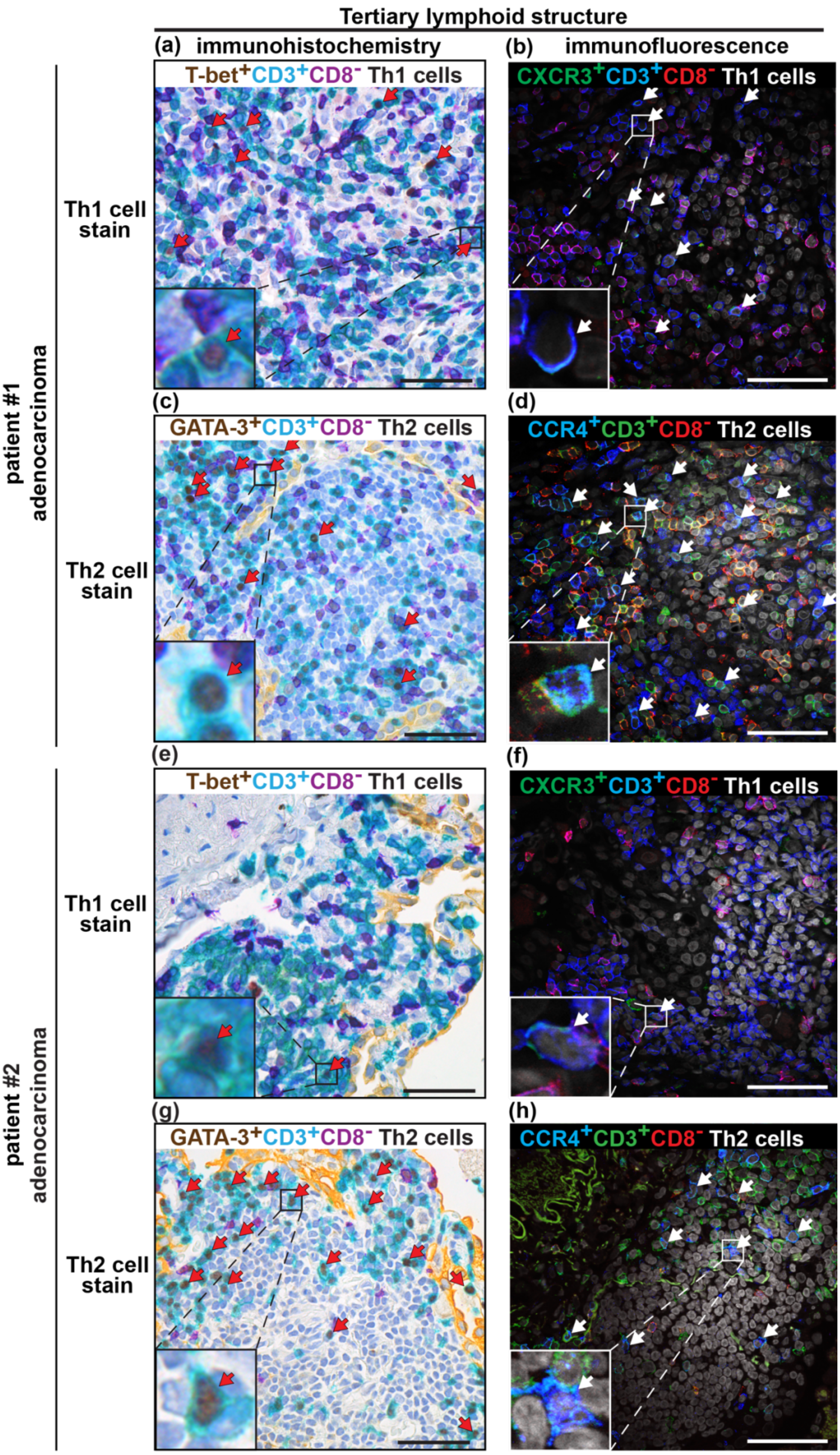

Supplementary Figure 10. Comparison of the number of Th1 and Th2 cells detected by chromogenic IHC vs. immunofluorescence in tertiary lymphoid structures (TLS). Representative images of immunostained sections of TLS from two patients with lung adenocarcinoma. Boxed areas are enlarged and presented as corner insets. The positive cells for each Th subset in tumor stroma are indicated by red or white arrows. **(a, e)** Th1 cells (T-bet$^+$CD3$^+$CD8$^-$) detected by chromogenic IHC. **(b, f)** Th1 cells (CXCR3$^+$CD3$^+$CD8$^-$) detected by immunofluorescence. **(c, g)** Th2 cells (GATA-3$^+$CD3$^+$CD8$^-$) detected by chromogenic IHC, using the anti-GATA-3 mAb clone L50-816. **(d, h)** Th2 cells (CCR4$^+$CD3$^+$CD8$^-$) detected by immunofluorescence. Similar results were observed in TLS from all the investigated NSCLC patients (n=3). Original magnification, 600x. Scale bars, 50 μm.

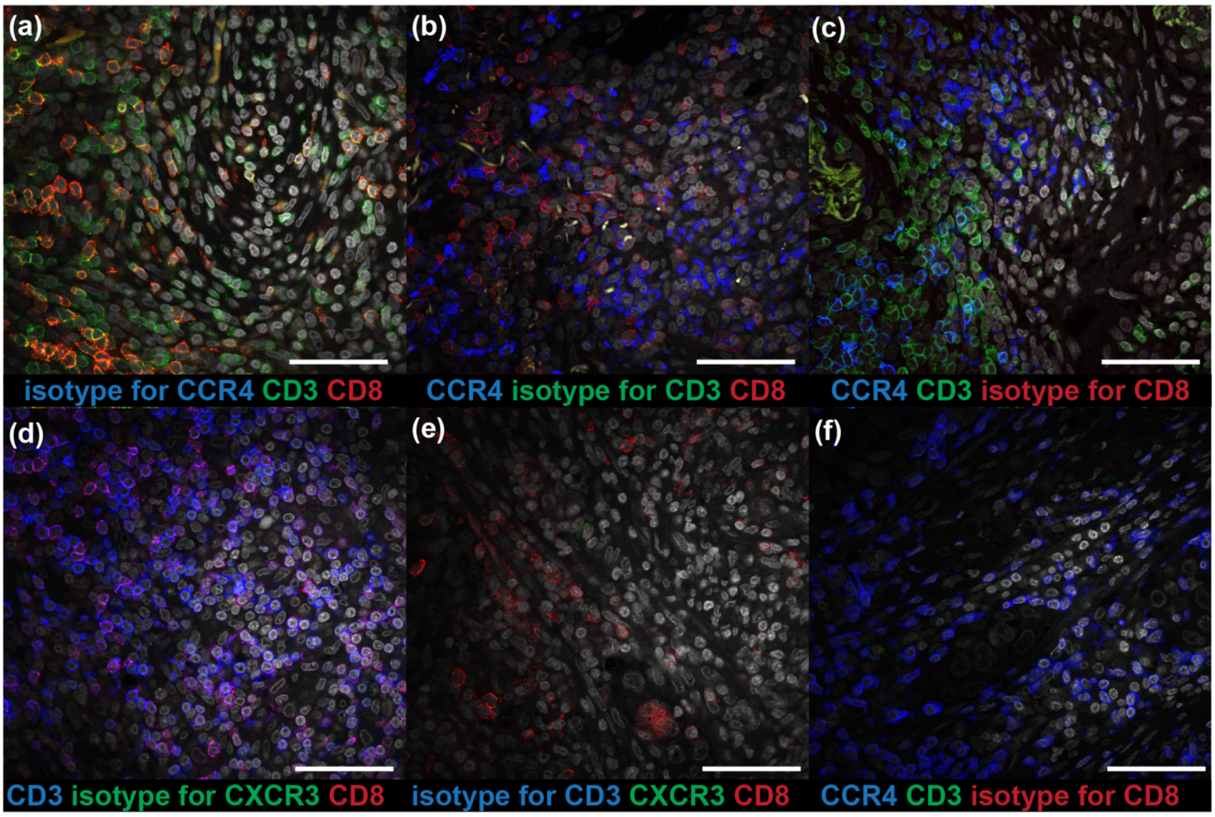

Supplementary Figure 11. Isotype-matched antibodies with irrelevant specificity as controls for immunofluorescence staining. (a-c) As controls for the CCR4/CD3/CD8 immunofluorescence staining, NSCLC tumor tissue sections were incubated with **(a)** isotype-matched control (rabbit IgG) for the CCR4 polyclonal antibody (blue) plus anti-CD3 mAb clone F7.2.38 (green) plus anti-CD8 mAb clone 4B11 (red); **(b)** anti-CCR4 polyclonal antibody (blue) plus anti-CD8 mAb clone 4B11 (red) plus isotype-matched control (mouse IgG1) for anti-CD3 antibody clone F7.2.38 (green); **(c)** anti-CCR4 polyclonal antibody (blue) plus anti-CD3 mAb clone F7.2.38 (green) plus isotype-matched control (mouse IgG2b) for anti-CD8 antibody clone 4B11 (red). **(d-f)** As controls for the CD3/CXCR3/CD8 immunofluorescence staining, tissue sections were incubated with **(d)** anti-CD3 mAb clone 2GV6 (blue) plus anti-CD8 mAb clone 4B11 (red) plus isotype-matched control (mouse IgG1) for the CXCR3 antibody clone 49801 (green); **(e)** anti-CXCR3 antibody clone 49801 (green) plus anti-CD8 mAb clone 4B11 (red) plus isotype-matched control (rabbit IgG) for anti-CD3 antibody (blue); **(f)** anti-CXCR3 antibody clone 49801 (green) plus anti-CD3 mAb clone 2GV6 (blue) plus isotype-matched control (mouse IgG2b) for anti-CD8 antibody clone 4B11 (red). Original magnification, 600x. Scale bars, 50 μm.
